# Altered methylation pattern in EXOC4 is associated with stroke outcome: an epigenome-wide association study

**DOI:** 10.1186/s13148-022-01340-5

**Published:** 2022-09-30

**Authors:** Natalia Cullell, Carolina Soriano-Tárraga, Cristina Gallego-Fábrega, Jara Cárcel-Márquez, Elena Muiño, Laia Llucià-Carol, Miquel Lledós, Manel Esteller, Manuel Castro de Moura, Joan Montaner, Anna Rosell, Pilar Delgado, Joan Martí-Fábregas, Jerzy Krupinski, Jaume Roquer, Jordi Jiménez-Conde, Israel Fernández-Cadenas

**Affiliations:** 1 Stroke Pharmacogenomics and Genetics, IIB-Sant Pau, Institut de Recerca de Sant Pau, Hospital Sant Pau, C/Sant Antoni Mª Claret,167, 08025 Barcelona, Spain; 2 Neurology, Hospital Universitari MútuaTerrassa/Fundacio Docència i Recerca MutuaTerrassa, Terrassa, Spain; 3 Facultat de Medicina, Universitat de Barcelona, Barcelona, Spain; 4 Neurology, Hospital del Mar, Neurovascular Research Group, IMIM, Universitat Autònoma de Barcelona/DCEXS‐Universitat Pompeu Fabra, Barcelona, Spain; 5 Department of Psychiatry, Washington University School of Medicine, Saint Louis, Missouri, USA; 6 NeuroGenomics and Informatics, Washington University School of Medicine, Saint Louis, Missouri, USA; 7 Cancer Epigenetics & Biology Program (PEBC), L’Hospitalet, Spain; 8 Department of Physiological Sciences II, School of Medicine, Universitat de Barcelona, Barcelona, Spain; 9 Institució Catalana de Recerca I Estudis Avançats (ICREA), Barcelona, Spain; 10 Neurovascular Research Laboratory, Vall d’Hebron Institut de Recerca (VHIR), Barcelona, Spain; 11 Department of Neurology, Hospital Universitario Virgen Macarena Sevilla, Instituto de Biomedicina de Sevilla, IBiS/Hospital Universitario Virgen del Rocío/CSIC, Universidad de Sevilla, Sevilla, Spain; 12 Neurology, Hospital de La Santa Creu i Sant Pau, Barcelona, Spain; 13 Centre for Bioscience, School of HealthCare Science, Manchester Metropolitan University, Manchester, UK

## Abstract

**Background and purpose:**

The neurological course after stroke is highly variable and is determined by demographic, clinical and genetic factors. However, other heritable factors such as epigenetic DNA methylation could play a role in neurological changes after stroke.

**Methods:**

We performed a three-stage epigenome-wide association study to evaluate DNA methylation associated with the difference between the National Institutes of Health Stroke Scale (NIHSS) at baseline and at discharge (ΔNIHSS) in ischaemic stroke patients. DNA methylation data in the Discovery (*n* = 643) and Replication (*n* = 62) Cohorts were interrogated with the 450 K and EPIC BeadChip. Nominal CpG sites from the Discovery (*p* value < 10^–06^) were also evaluated in a meta-analysis of the Discovery and Replication cohorts, using a random-fixed effect model. Metabolic pathway enrichment was calculated with methylGSA. We integrated the methylation data with 1305 plasma protein expression levels measured by SOMAscan in 46 subjects and measured RNA expression with RT-PCR in a subgroup of 13 subjects. Specific cell-type methylation was assessed using EpiDISH.

**Results:**

The meta-analysis revealed an epigenome-wide significant association in *EXOC4* (*p* value = 8.4 × 10^–08^) and in *MERTK* (*p* value = 1.56 × 10^–07^). Only the methylation in *EXOC4* was also associated in the Discovery and in the Replication Cohorts (*p* value = 1.14 × 10^–06^ and *p* value = 1.3 × 10^–02^, respectively). *EXOC4* methylation negatively correlated with the long-term outcome (coefficient = − 4.91) and showed a tendency towards a decrease in *EXOC4* expression (rho = − 0.469, *p* value = 0.091). Pathway enrichment from the meta-analysis revealed significant associations related to the endocytosis and deubiquitination processes. Seventy-nine plasma proteins were differentially expressed in association with *EXOC4* methylation. Pathway analysis of these proteins showed an enrichment in natural killer (NK) cell activation. The cell-type methylation analysis in blood also revealed a differential methylation in NK cells.

**Conclusions:**

DNA methylation of *EXOC4* is associated with a worse neurological course after stroke. The results indicate a potential modulation of pathways involving endocytosis and NK cells regulation.

**Supplementary Information:**

The online version contains supplementary material available at 10.1186/s13148-022-01340-5.

## Introduction

Stroke is a high incidence disease that represents the first cause of death and disability in adults [1, 2]. More than 70% of stroke survivors need help for their daily activity 5 years after an ischaemic stroke [3].

During the acute phase of a stroke, there are dynamic changes in the clinical symptoms that determine the evolution of the lesion and the associated deficits [4]. To measure the outcome of a stroke, two different quantifiable measures are usually considered: the neurological clinical symptoms and the functional independence of patients. There is high variability in the neurological and functional outcomes after stroke which is associated with several factors, including demographic, clinical and genetic factors [5–7]. Different scales are widely used to quantify the neurological deficit and the functional outcome. The National Institutes of Health Stroke Scale (NIHSS) considers 15 different measures of neurological worsening: consciousness, eye movement, vision, coordination, language, sensory function, upper and lower limb strength, facial muscle function, and neglect [8]. The modified Rankin scale (mRS) is used to quantify the functional outcome based on the capacity of patients to be independent in carrying out daily activities [9]. Different variables have been found to be predictors of the early (24 h post-stroke) neurological outcome: baseline NIHSS, tPA treatment, age, stroke subtype, glucose levels, and systolic blood pressure [5–7]. The early neurological outcome has been found to explain up to 30% of the long-term outcome (mRS at 3 months) [5]. The neurological evolution of stroke patients during hospitalization has also been assessed as a good predictor of 30-day and long-term mortality [10]. Reznik et al*.* compared the predictive value of NIHSS measured at different time-points: baseline, 24 h, and discharge. They concluded that discharge-NIHSS was the best predictor of the 3-month outcome [11]. Thus, the difference between the NIHSS at baseline and the NIHSS at discharge has recently gained importance as a valid outcome variable [12] as it covers the entire period of hospitalization.

Interestingly, genetics seems to play a role in the neurological course. Ibanez L et al*.* found that 8.7% of the difference between NIHSS at baseline and NIHSS at 24 h was explained by common single nucleotide polymorphisms (SNPs) [13]. However, only three different Genome-Wide Association Studies (GWAS) have been performed in the stroke outcome field [13–15]. Two genes (*PATJ* and *LOC105372028*) have been associated with long-term functional outcome (at 3 months) [14, 15] and seven loci with the neurological course using the difference between baseline NIHSS and NIHSS at 24 h [13]. However, not all the heritability associated with the neurological course has been completely discovered [13] and other heritable factors, such as epigenetics, could be associated with the post-stroke neurological outcome. It has been demonstrated that epigenetics plays an important role in stroke risk and stroke vascular recurrence [16–21]. Previous Epigenome-Wide Association Studies (EWAS) have identified 22 CpG sites and 21 loci with altered DNA methylation associated with stroke risk [16]. Moreover, biological age calculated with DNA methylation is associated with stroke outcome and mortality [20, 21].

Our aim is to study the epigenetic risk factors and biological mechanisms associated with post-stroke neurological course using the difference in baseline NIHSS and NIHSS at discharge (ΔNIHSS) as outcome variable.

## Materials and methods

### Data availability

The DNA methylation data analysed in this study are available in GEO. The Discovery data from BASICMAR are available under the GEO accession number “GSE69138”. The Discovery from the GRECOS together with the replication cohort could be identified in GEO under the code “GSE203399”.

### Patient selection

We included in the Discovery Cohort 738 Caucasian patients with EWAS data who had suffered a stroke and had had a blood sample taken during the first 24 h following ischaemic stroke. The Discovery consisted in patients from the Mar Hospital, who enrolled 662 patients as part of the BASICMAR register [22–24], and 76 from the GRECOS study [25]. BASICMAR is a prospective register of patients with ischaemic stroke recruited between 2009 and 2012 [22–24]. The GRECOS (Genotyping RECurrence Risk of Stroke) study is a project that enrolled 1,494 Caucasian patients with a first ischaemic stroke and population-based controls between July 2005 and May 2009 from 23 Spanish Hospitals [25]. The patients included from the BASICMAR and the GRECOS study were included in previous EWAS [16, 18, 19]. From the 738 patients, 725 had registered the main variable analysed in this study: the NIHSS at baseline and the NIHSS at discharge and were included in the EWAS analysis.

In the Replication Cohort, we included 62 Caucasian stroke patients from the EPIGENESIS study. The EPIGENESIS study selected ischaemic stroke patients [26] with a blood sample collected during the first 6 h following onset of symptoms to study epigenetics associated with stroke outcome.

Differences in demographic and clinical variables between the Discovery and Replication cohorts were calculated. Differences between groups were tested with Kruskal–Wallis rank-sum test for nonparametric quantitative variables, while differences for qualitative variables was tested with chi-square test. Statistically significance was defined with *p* value < 0.05.

All the projects included in this study have been approved by ethics committees and all the patients have signed informed consent forms. The study was conducted in accordance with Declaration of Helsinki and European guidelines: requirements of the Spanish Law 3/18 on the protection of personal data and the new European Union legislation on personal data, specifically Regulation (EU) 2016/679 of the European Parliament and of the Council of April 27, 2016 Data Protection (GDPR).

### DNA extraction and bisulphite conversion

Whole blood was obtained in EDTA tubes. DNA from the GRECOS and EPIGENESIS studies was extracted using a Gentra Puregene Blood Kit (Qiagen, Hilden, Germany) following the manufacturer’s instructions. DNA from BASICMAR was extracted manually using salt precipitation in the National Bank of DNA (Carlos III Institute (ISCIII)).

Bisulphite conversion of DNA was performed before EWAS analysis using the EZ DNA Methylation-Gold™ Kit (Zymo Research, CA, USA). DNA methylation was studied with the 450 K BeadChip (Illumina) in all samples from the Discovery Cohort and EPIC BeadChip (Illumina) in all samples from the Replication Cohort.

### Epigenome-wide association study (EWAS)

Methylation raw data were processed using R (http://www.cran.r-project.org) and Bioconductor packages (http://www.bioconductor.org).

We proceed with quality controls (QCs) using ChAMP package in *R* [27]. CpG quality controls consisted of the removal of CpG sites with a non-significant detection *p* value (*p* value > 0.05), CpG sites from sex chromosomes, CpG sites with affinity for multiple probes, no “CG” probes, probes with bead count < 3 in at least 5% of samples and SNP probes [28]. When performing QCs on samples, we removed samples with more than 1% missing CpG sites and samples with discordance between genotypic and phenotypic sex (Additional file 1: Figure SI). After this processing, beta values representing methylation of each CpG site were normalized using the Noob function from the minfi package [29]. We also assessed the batch effect by a single value decomposition (SVD) analysis in ChAMP and by performing a multidimensional scaling (MDS) plot where it is shown the distance matrix of each sample depending on the batch (Additional file 1: Figure SII). The proportion of the different blood cell types was estimated for each patient, and beta values were corrected based on these data using the “champ.refbase” function of the ChAMP package [30]. This function has implemented the RefbaseEWAS method, which uses a methylation reference database for each of the major cell types present in blood.

### Statistical analysis

We calculated the difference between baseline NIHSS and NIHSS at discharge (ΔNIHSS). First, we evaluated which demographic, cardiovascular and other stroke-related variables were associated with ΔNIHSS. We also assessed whether ΔNIHSS and other variables were associated with the long-term outcome (measured with the Rankin scale at 3 months, mRS) in our cohort using bivariate analysis. Then, we included the significant variables (*p* value < 0.05) in a backward stepwise regression.

The DNA methylation was considered the dependent variable and the ΔNIHSS the independent variable. We calculated the differential methylation positions using multiple linear regression (lm). First, in the Discovery analysis we considered as covariates the known variables to be associated with the dependent variable (DNA methylation): sex, age, self-reported smoking habits, and the first two principal components (PC) (basic EWAS adjustment). For the PC calculation, we used the function *princomp* in R from stats package which uses a spectral decomposition strategy to study the correlation between the methylation beta values. We selected the first two columns from the loading matrix (eigenvectors) from the output to adjust the results. To ensure that the batch effect was corrected when adjusting by the first two PC, we performed an additional analysis adjusting in the lm by the basic EWAS adjustment + batch. We used the MethylToSNP package in R to evaluate whether any of the CpG sites with a *p* value < 0.05 had SNP patterns and was not removed during QCs that could cause false positive results [31].

As a secondary analysis, we considered the ΔNIHSS as a dichotomic variable, classifying patients into those with improvement in the outcome (ΔNIHSS ≥ 4) or decline in the outcome (ΔNIHSS < 4), based on previous studies [32, 33]. The purpose was to assess whether nominal CpG sites associated with the continuous ΔNIHSS variable were also associated with neurological improvement or decline.

**Fig. 1:**
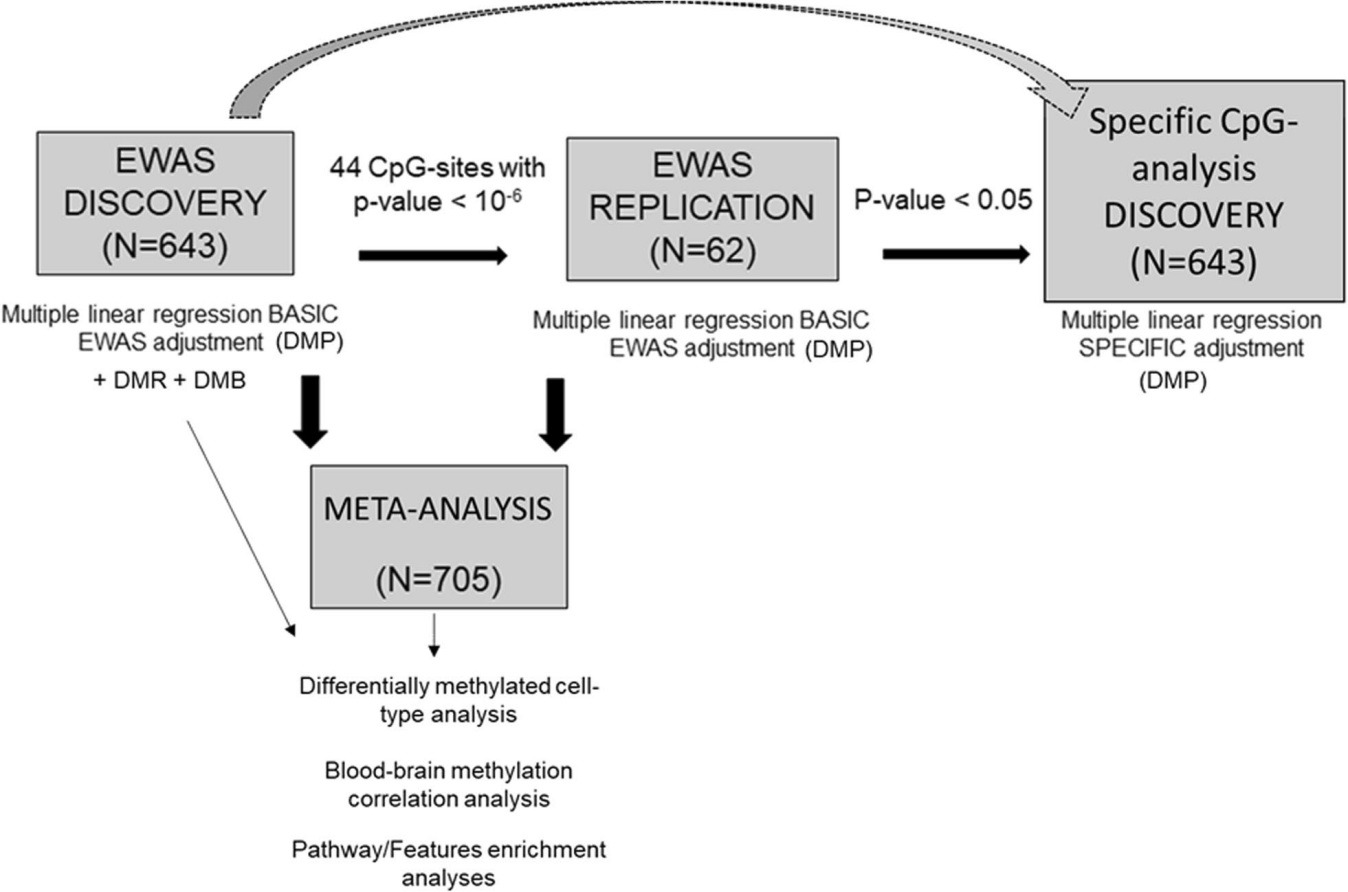
Scheme of the three-stage EWAS. The three-stage EWAS consisted in a Discovery stage analysed with multiple linear regression with adjustment of variables known to be associated with methylation (age, sex, smoking habit, PC1 and 2), a Replication stage where the 44 nominally associated CpG sites were analysed with multiple linear regression with the same basic adjustment than in the Discovery. The replicated CpG site (cg00039070) was re-analysed in the Discovery including the specific adjustment (covariates associated with the methylation of cg00039070): PC1 and PC2. In the Discovery cohort, differentially methylation region (DMR) and block (DMB) analyses were also performed. Finally, a meta-analysis that merged the Discovery and Replication Cohorts was performed with basic EWAS adjustment. The significant CpG sites in the meta-analysis were evaluated in the pathway/features enrichment analysis, in the differentially methylated cell-type analysis and in the blood–brain correlation analysis

In the Discovery Analysis, all the significant, with a *p* value < 2.4 × 10^–07^ (based on the threshold estimated by Saffari A et al. [34, 35]), and nominal (*p* value < 10^–06^) differentially methylated positions (DMP) were analysed in the Replication Cohort (Replication Analysis). The CpG methylation sites with *p* value < 0.05 in the Replication Cohort were considered replicated. The methylation from the replicated CpG sites were analysed in bivariate and backward stepwise regression analyses to identify independent clinical and demographic factors conditioning the methylation pattern of that replicated sites. Finally, the independent variables were used as covariates in a new lm analysis including only the replicated CpG sites in the Discovery Cohort (Fig. 1).

Moreover, we combined the results from the Discovery and Replication Cohorts in a meta-analysis using METAL [36] based on a random effect model. We selected this model after reviewing the assumptions of random and fixed effect models [37]. Based on Nikolakopoulou A et al*.*, the random effect model could be beneficious on allowing to differ on the true effects and accounting for unexplained heterogeneity [37]. The association of the nominally CpG sites from the Discovery was evaluated in the meta-analysis.

Finally, we considered all the CpG sites that followed the next criteria as validated DMPs: 1) at least a nominal association (*p* value < 10^–06^) in the Discovery Cohort, 2) significant *p* value (*p* value < 0.05) and the same effect direction in the Replication Cohort, and 3) an epigenome-wide significant *p* value (*p* value < 2.4 × 10^–07^) in the meta-analysis.

### Feature enrichment and metabolic pathway enrichment analyses

We explored for enrichment of specific features for significant (*n* = 5) or nominal (*n* = 44) CpG sites from the discovery in comparison with all the CpG sites included in the analysis (*n* = 423,156). Features were classified into 1st exon, 3’ untranslated region (UTR), 5’UTR, gene body, intergenic region (IGR), transcription start site (TSS) 1500 and TSS200. In this analysis, we classified CpG sites into hypermethylated or hypomethylated in their association with ∆NIHSS. We applied chi-square test to evaluate differences in the features between groups (significant/nominal CpG sites vs. all CpG sites).

We looked for enrichment of metabolic pathways using as input all the CpG sites and the CpG sites that were nominally associated (*p* value < 10^–06^) with ΔNIHSS in the meta-analysis. The analysis was done with MethylGSA [38], an R package specifically designed for pathway analysis from EWAS results. We applied the three functions (methylglm, methylRRA and methylgometh) from the package (all of them designed to adjust for the number of CpGs in each gene to reduce possible bias). In the main analysis, all the CpG sites from the EWAS meta-analysis were included together with their *p* value. Using the first and second function, we also restricted the analysis to specific types: CpGs from promoters (TSS1500 or TSS200) or located in gene bodies. Using the second and third function, we were able to perform a secondary analysis filtering out CpG sites based on their *p* value, selecting the list of nominally associated CpG sites from the meta-analysis. More details for the different functions and the options that we selected are specified in the Supplementary methods. We included in the analyses the three available pathway databases in MethylGSA: Gene Ontology, KEGG and Reactome. We considered significant associations when the *p* value was < 0.05 and the Q value (false discovery rate (FDR) adjusted *p* value) was < 0.05.

### Differentially methylated regions (DMRs) and blocks (DMBs)

We evaluated whether differential methylation regions (DMRs) and blocks (DMBs) were associated with ΔNIHSS using the Bumpunther algorithm implemented in the ChAMP package [27, 30]. A DMR was defined in our analysis with the standard parameters: a segment with a minimum of seven CpG sites with a maximum length of 300 nucleotides. DMRs should be separated by a minimum of 1,000 base-pairs to be considered different DMRs. For the DMB calculation, a block was considered a large cluster generated from open sea regions. Each region located in an open sea was collapsed into a unit, calculating the mean methylation and mean position. We used the standard recommendation of a maximum length of 250,000 nucleotides for a block and to only include blocks with a minimum of 10 regions [27, 30].

### Gene expression analysis

We studied the correlation between relative *EXOC4* mRNA levels and *EXOC4* methylation. We analysed by Real-Time Quantitative Reverse Transcription PCR (qRT-PCR) the cDNA isolated from whole blood from 13 healthy controls from the GRECOS study which also have available EWAS data.

Whole blood in EDTA tubes was obtained, and DNA was extracted using a Gentra Puregene Blood Kit (Qiagen, Hilden, Germany) following the manufacturer’s instructions. We used the 7900 Real-Time PCR system (qRT-PCR) (Applied Biosystems, Foster city, CA, USA) to quantify *EXOC4* expression. We followed a standard TaqMan® PCR kit protocol as described previously [15]. Briefly, the *EXOC4* (Hs00253986_m1) probe was used and the results were normalized using endogenous controls: Cyclophilin A (*PPIA*, Hs99999904_m1) and Glyceraldehyde-3-Phosphate Dehydrogenase (*GAPDH*, Hs99999905_m1). We ran triplicates for the 13 samples, and we included an external sample as calibrator. Reactions were analysed with applied Biosystems SDS 7900 system software (Applied Biosystems, Foster city, CA, USA). We calculated the fold change of the average expression (using relative quantification (RQ) values) from *EXOC4* and endogenous controls.

### Proteomic and pathway analysis

We used data from SOMAscan® Assay (SomaLogic) to find differentially expressed proteins associated with cg00039070 methylation. Briefly, the SOMAscan® Assay uses plasma samples to bring data for 1305 proteins using a short single-stranded DNA sequence (SOMAmer reagents) that binds to target proteins and allows their quantification [39].

In this analysis, we included 26 stroke patients and 20 controls for which proteomic (from SOMAscan®) and DNA methylation data (from EWAS) were available. For the proteomic assay, blood from patients collected in EDTA tubes was centrifuged at 3.000 g for 10 min to obtain plasma. Plasma samples were frozen at − 80 °C until they were analysed with SOMAscan®. The results were processed as described in [40]. The analysis was adjusted by case/control state.

We investigated differentially expressed proteins in association with cg00039070 methylation. Stroke patients were included from the EPIGENESIS (*N* = 7) and GRECOS (*N* = 19) cohorts. Controls were included from the ISSYS (Investigating Silent Stroke in hYpertensives) cohort. It is an observational prospective study in hypertensive participants to determine the prevalence of silent or magnetic resonance imaging (MRI)-defined brain infarcts and cognitive impairment. This cohort comprises 1000 non-demented individuals, aged 50 to 70 years, and diagnosed with essential hypertension at least one year before inclusion in the ISSYS study [41].

All the proteins associated with cg00039070 methylation (*p* value < 0.05) were analysed using over-representation analysis (ORA) to find enrichment of metabolic pathways using the Wilcoxon Rank-Sum Test in the Gene Ontology database (Biological Process) in WebGestalt [42, 43].

### Differentially methylated cell-type (DMCT) analysis

The cellular component of the blood tissue was estimated and analysed to determine whether the differential methylation was specific to one cell type using EpiDISH [44]. The CellDMC function was used to identify differentially methylated cell types associated with the ΔNIHSS based on the proportion of B cells, CD4 + and CD8 + T cells, NK, neutrophils and monocytes. We studied the nominally associated CpG sites in the meta-analysis to find enrichment of cell-type differential methylation.

### Tissue-specific signal detection

We used eFORGE (experimentally derived Functional element Overlap analysis of ReGions from EWAS) [45] to estimate tissue-specific signals from the significant and nominal results from the EWAS meta-analysis. We included analyses with all the different functional elements. A *p* value < 0.05 was considered a nominal association and a *Q* value < 0.05 according to FDR adjustment was considered statistically significant. A more complete description of this tool is provided in Supplemental Methods (Additional file 1).

### Blood–brain epigenetic correlation

In order to compare the methylation from the significant findings in the meta-analysis between blood and brain, we used the Blood Brain DNA Methylation Comparison Tool [46]. We analysed the correlation of cg00039070 methylation and the four brain regions included in the tool.

Using *Blood–Brain Epigenetic Concordance* (BECon) [47], we also investigated the concordance in the cg00039070 methylation between three brain regions (Brodmann area (BA) 10, BA20 and BA7) and blood using the three metrics available in this tool.

A more detailed explanation for these two methods is included in the Supplemental Methods (Additional file 1).

## Results

### Discovery stage

A total of 643 patients and 423,156 CpG sites passed QCs in the Discovery analysis (Additional file 1: Figure SI). The median NIHSS at baseline and discharge was 8 and 3, respectively, and the median ΔNIHSS at discharge was positive, indicating an improvement in the neurological status of these patients (Table 1). We wanted to assess which variables were independently associated with the ΔNIHSS in our cohort. With this purpose, we studied the association of different demographic, cardiovascular and stroke-related variables, including the long-term outcome (measured with the Rankin scale at 3 months) with ΔNIHSS. Then, we explored which variables were independently associated with the mRS, to be sure that the main variable in our analysis (ΔNIHSS) was a good predictor of long-term outcome in our cohort. We performed bivariate and backward stepwise regression analyses. NIHSS at baseline (*p* value < 2.2 × 10^–16^), mRS (*p* value < 2.2 × 10^–16^), treatment with rTPA (*p* value = 2.5 × 10^–02^) and atrial fibrillation (*p* value = 1.75 × 10^–02^) were independently associated with ΔNIHSS in a multivariate analysis (Additional file 1: Table SI), whereas ΔNIHSS (*p* value < 2.2 × 10^–16^), baseline NIHSS (*p* value < 2.2 × 10^–16^), age (*p* value = 4.72 × 10^–10^), sex (*p* value = 1.3 × 10^–02^) and smoking habit (*p* value = 4.4 × 10^–02^) were found to be associated with the 3 months of mRS in the stepwise regression analysis (Additional file 1: Table SII). ΔNIHSS at discharge and mRS at 3 months were negatively correlated, indicating that a worsening in the neurological course measured with ΔNIHSS was associated with a worse long-term outcome measured with mRS.

**Table 1:** Demographic and clinical data from the Discovery and Replication Cohorts

	Discovery		Replication	*P* value
	GRECOS	BASICMAR		
*Subjects(n)*	59	584	62	
Sex				0.429
Male,* n* (%)	49 (17%)	322 (55.2%)	32 (51.6%)	
Female,* n* (%)	10 (83%)	262 (44.8%)	30 (48.4%)	
Age in years, median (IQR)	71 (15)	77 (14)	77 (9.5)	0.22
NIHSS at baseline, median (IQR)	3 (6.5)	5 (8)	15 (11.5)	< 2.2 × 10^–16*^
NIHSS at discharge, median (IQR)	1 (2)	3 (6)	4 (12)	2.62 × 10^–06*^
ΔNIHSS, median (IQR)	1 (4)	1 (4)	5 (11)	6.6 × 10^–04*^
mRS 90 days, median (IQR)	1 (3)	2 (3)	1 (3)	5.4 × 10^–04*^
Presence of HTN, * n* (%)	37 (62.7%)	236 (40.1%)	40 (64.5%)	1.04 × 10^–03*^
Smoking, * n* (%)	13 (22%)	168 (28.8%)	6 (9.67%)	3 × 10^–07*^
Presence of AF, * n* (%)	3 (5%)	201 (34.4%)	25 (40.3%)	2.86 × 10^–02*^
Presence of DM, * n* (%)	17 (28.8%)	421 (72.1%)	8 (12.9%)	9.5 × 10^–11*^
Treatment with rtPA, * n* (%)	9 (15.3%)	91 (15.6%)	53 (85.5%)	< 2.2 × 10^–16*^
TOAST				
CES,* n* (%)	0 (0%)	231 (53.9%)	29 (46.7%)	< 2.2 × 10^–16*^
LAS, * n* (%) SVS, * n* (%) Und, * n* (%) Other, * n* (%) NA	26 (44.1%) 11 (18.6%) 21 (35.6%) 1 (1.7%) 0 (0%)	153 (39.6%) 194 (33.2%) 4 (0.7%) 2 (0.34%) 0 (0%)	17 (27.4%) 0 (0%) 13 (2.1%) 0 (0%) 3 (4.8%)	

Main clinical characteristics of patients included in the analysis from the Discovery and Replication Cohorts.* P* value column indicates differences for each variable between Discovery (both GRECOS and BASICMAR together) and Replication Cohorts

*IQR* interquartile range; ΔNIHSS = NIHSS at baseline – NIHSS at discharge;* HTN:* hypertension;* AF* atrial fibrillation; DM: diabetes mellitus;* CES* cardioembolic stroke;* LAS* large artery stroke;* SVS* small vessel stroke;* und* undetermined stroke

*Statistically significant p values for differences between cases and controls. NA: not available

ΔNIHSS was used as the independent variable to calculate differential methylation positions using multiple linear regression (lm) (Fig. 1).

**Fig. 2:**
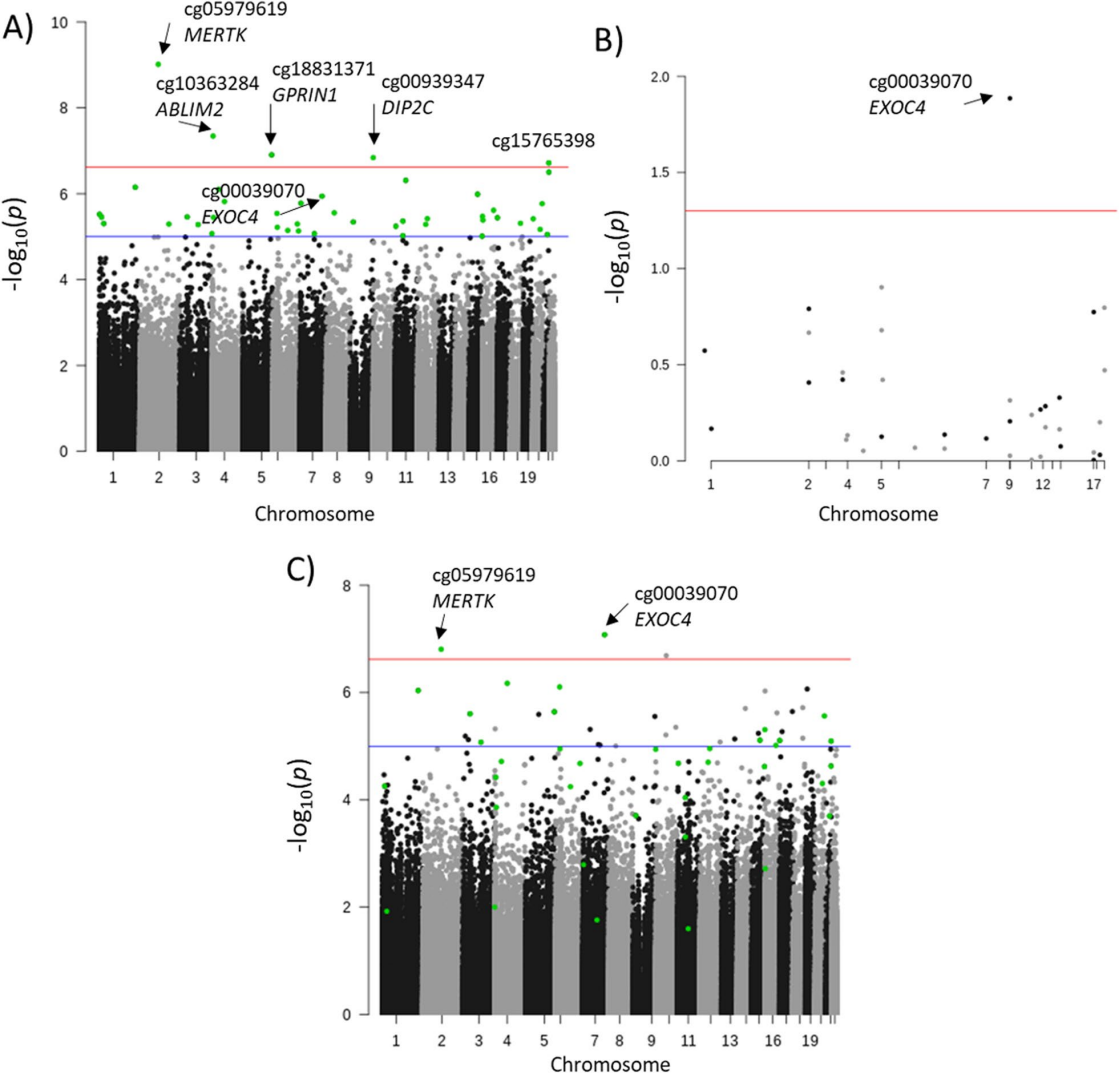
Manhattan plots for the EWAS analyses. Manhattan plot in the Discovery Analysis (**A**), Replication Analysis (**B**) and meta-analysis (**C**). The green dots represent CpG sites nominally associated with ΔNIHSS in the Discovery Cohort. The red and blue lines represent the epigenome-wide and nominal significance threshold, respectively. CpG sites are annotated in the plot if they are nominally associated with NIHSS in the Discovery Cohort and are statistically significant in the corresponding analysis

In the Discovery EWAS with basic adjustment (sex, age, smoking habits and the first two PCs), we identified a total of five epigenome-wide (*p* value < 2.4 × 10^–07^) CpG sites and 44 nominally CpG sites (*p* value < 10^–06^) associated with the ΔNIHSS (Fig. 2, Table 2). When the batch variable (defined by the two cohorts included in the discovery) was used as covariate, the results did not change remarkably, and all the 44 CpG sites remained significant with a *p* value < 0.05 (Additional file 1: Table SIII). Thus, the observed batch effect (Additional file 1: Figure SII) was corrected when adjusting by PCs. Using MethylToSNP, none of the evaluated CpG sites was predicted to have SNP patterns.

**Table 2:** EWAS summary statistics in the Discovery, Replication and meta-analyses

					Discovery		Replication		Meta-analysis		
CpG	CHR	BP	Gene	Feature	COEFF (UCI,LCI)	*P*	COEFF (UCI,LCI)	*P*	COEFF (UCI,LCI)	*P*	Direction
**cg00039070**	7	133,482,303	*EXOC4*	Body	− 4.91(− 6.86,− 2.96)	1.14E-06	− 2.58(− 4.62,− 0.54)	1.30E-02	− 5.474(− 5.4738,− 5.4742)	**4.44E-08**	− −
**cg05979619**	2	111,947,000	*MERTK*	Body	− 6.21(− 8.16,− 4.26)	9.72E-10	− 1.26(− 3.24,0.72)	2.15E-01	− 5.250(− 5.2495,− 5.2501)	**1.54E-07**	− −
cg10977834	4	77,075,687	*CCNI*	5'UTR	4.85(6.80,2.90)	1.53E-06	1.56(3.55,− 0.43)	1.25E-01	4.875(4.8749,4.8747)	*1.11E-06*	+ +
cg08119231	1	220,747,874	*MOSC2*	TSS1500	5.01(6.96,3.06)	7.07E-07	0.87(2.83,− 1.09)	3.91E-01	4.871(4.8717,4.8704)	1.11E-06	+ +
cg21574204	6	28,081,180	*ZNF165*	5'UTR	4.72(6.67,2.77)	2.88E-06	1.27(3.24,− 0.70)	2.09E-01	4.847(4.8470,4.8470)	*1.21E-06*	+ +
cg01149259	3	46,979,906	*NBEAL2*	1stExon	4.68(6.63,2.73)	3.48E-06	1.42(3.41,− 0.57)	1.62E-01	4.768(4.7684,4.7682)	*1.86E-06*	+ +
cg18831371	5	176,610,324	*GPRIN1*	TSS200	5.35(7.30,3.40)	1.25E-07	0.32(2.17,− 1.53)	7.48E-01	4.752(4.7527,4.7522)	2.02E-06	+ +
cg26050512	20	58,981,011	*TH1L*	TSS1500	4.83(6.78,2.88)	1.71E-06	− 0.09(− 1.93,1.75)	9.30E-01	4.563(4.5626,4.5624)	4.83E-06	− +
cg21404878	15	72,286,264	*BRUNOL6*	3'UTR	4.94(6.43,2.51)	1.03E-06	0.02(2.19,− 2.15)	6.69E-01	4.540(4.5407,4.5393)	5.61E-06	+ +
cg09548897	16	291,277	*AXIN1*	Body	− 4.69(− 6.64,− 2.74)	3.37E-06	− 0.73(− 2.67,1.21)	4.69E-01	− 4.499(− 4.4986,− 4.4989)	6.80E-06	− −
cg14659930	3	114,409,454	*ZBTB20*	5'UTR	4.59(6.48,2.58)	5.24E-06	0.19(2.08,− 1.70)	3.78E-01	4.453(4.4544,4.4511)	8.48E-06	+ +
cg00939347	10	606,320	*DIP2C*	Body	− 5.32(− 7.27,-3.37)	1.45E-07	− 0.5(− 2.42,1.42)	6.22E-01	− 4.450(− 4.4501,− 4.4506)	8.50E-06	− −
cg07925823	16	68,264,601	*SLC7A6*	5'UTR	4.76(6.71,2.81)	2.43E-06	1.4(3.39,− 0.59)	1.68E-01	4.443(4.4430,4.4428)	8.81E-06	+ +
cg20383948	21	45,478,223	*COL18A1*	Body	5.17(7.12,3.22)	3.15E-07	0.97(2.93,− 0.99)	3.38E-01	4.431(4.4307,4.4305)	9.28E-06	+ +
cg09741713	12	62,078,134	*FAM19A2*	5'UTR	4.66(6.63,2.73)	3.82E-06	− 1.12(− 3.08,0.84)	5.20E-01	4.340(4.3408,4.3393)	1.42E-05	+ +
cg12103149	6	30,213,396	*TRIM26*	TSS200	4.56(6.58,2.68)	6.06E-06	0.08(1.98,− 1.82)	3.79E-01	4.331(4.3312,4.3311)	1.45E-05	+ +
cg10156941	17	1,563,002	*PITPNA*	TSS200	4.67(6.62,2.72)	3.63E-06	0.12(1.90,− 1.66)	9.03E-01	4.349(4.3489,4.3488)	1.46E-05	+ +
cg15765398	21	44,990,079		IGR	− 5.27(6.51,2.61)	1.92E-07	0.89(2.85,− 1.07)	1.60E-01	− 4.328(− 4.3267,− 4.3288)	1.51E-05	+ -
cg12349416	11	4,184,868		IGR	4.58(6.89,2.99)	5.73E-06	0.43(2.32,− 1.46)	4.84E-01	4.321(4.3219,4.3207)	1.55E-05	+ +
cg14482313	12	52,233,105	*KRT7*	TSS200	4.60(6.55,2.65)	5.19E-06	0.62(2.55,− 1.31)	5.40E-01	4.304(4.3047,4.3037)	1.68E-05	+ +
cg02996131	6	152,637,463	*SYNE1*	TSS1500	4.60(6.61,2.71)	5.08E-06	0.65(2.58,− 1.28)	8.63E-01	4.252(4.2532,4.2516)	2.11E-05	+ +
cg19935850	4	41,751,422		IGR	4.99(6.94,3.04)	7.94E-07	− 0.14(− 1.91,1.63)	8.86E-01	4.182(4.1825,4.1808)	2.90E-05	− +
cg10363284	4	8,005,411	*ABLIM2*	Body	− 5.54(-7.49,− 3.59)	4.55E-08	0.29(2.18,− 1.60)	7.76E-01	− 4.179(− 4.1782,− 4.1790)	2.94E-05	+ −
cg18707780	15	99,733,585	*LYSMD4*	TSS200	4.46(6.41,2.51)	9.75E-06	− 0.41(− 2.31,1.49)	6.85E-01	4.154(4.1539,4.1538)	3.42E-05	− +
cg20648899	6	93,416,423	*EPHA7*	Body	4.53(6.60,2.70)	7.17E-06	− 0.2(− 2.03,1.63)	8.54E-01	4.007(4.0083,4.0057)	6.14E-05	+ +
cg07987148	20	46,690,251	*TP53RK*	TSS1500	4.54(6.56,2.66)	6.79E-06	− 0.41(− 2.28,1.46)	9.89E-01	3.980(3.9813,3.9790)	6.88E-05	- +
cg07475390	1	14,114,109		IGR	4.68(6.53,2.63)	3.52E-06	0.7(2.62,− 1.22)	2.67E-01	3.959(3.9593,3.9580)	7.55E-05	- +
cg00347584	11	47,261,474	*NR1H3*	Body	4.47(6.47,2.57)	9.50E-06	− 1.26(− 3.24,0.72)	9.87E-01	3.909(3.9098,3.9081)	9.27E-05	+ +
cg13114315	21	36,174,169	*DOPEY2*	Body	4.48(6.43,2.53)	8.92E-06	0.49(2.41,− 1.43)	6.30E-01	3.843(3.8438,3.8418)	1.22E-04	+ +
cg14414100	9	19,547,532	*SLC24A2*	Body	4.63(6.59,2.69)	4.55E-06	− 0.56(− 2.46,1.34)	9.40E-01	3.772(3.7733,3.7717)	1.62E-04	+ +
cg18795809	4	10,456,907	*ZNF518B*	5'UTR	4.68(6.63,2.73)	3.58E-06	0.34(2.22,− 1.54)	7.36E-01	3.771(3.7708,3.7703)	1.64E-04	+ +
cg03732020	11	47,261,417	*NR1H3*	Body	4.64(6.49,2.59)	4.35E-06	− 0.01(− 1.29,1.27)	5.76E-01	3.551(3.5523,3.5506)	3.83E-04	− +
cg24978805	7	3,687,557	*SDK1*	Body	− 4.84(− 6.79,− 2.89)	1.67E-06	− 0.35(− 2.24,1.54)	7.30E-01	− 3.184(− 3.1840,− 3.1845)	1.45E-03	− −
cg08526825	16	2,752,228	*SRRM2*	TSS200	4.65(− 7.22,− 3.32)	4.13E-06	1.43(3.42,-0.56)	8.41E-01	3.051(3.0506,3.0504)	2.28E-03	− +
cg25354926	4	818,823	*CPLX1*	Body	− 4.49(− 6.44,− 2.54)	8.52E-06	0.95(2.91,− 1.01)	3.47E-01	− 2.784(− 2.7840,− 2.7844)	5.37E-03	+−
cg04886221	1	27,343,238	*SYTL1*	5'UTR	4.61(6.55,2.65)	4.97E-06	0.17(1.97,− 1.63)	6.80E-01	2.532(2.5320,2.5319)	1.14E-02	− +
cg22363670	7	86,643,853	*GRM3*	TSS200	4.49(6.44,2.54)	8.55E-06	− 0.3(− 2.16,1.56)	7.65E-01	2.409(2.4095,2.4092)	1.59E-02	- +
cg06933752	11	65,122,316	*FAU*	TSS200	5.08(7.03,3.13)	4.90E-07	− 0.06(− 1.80,1.68)	9.51E-01	2.199(2.1995,2.1994)	2.80E-02	− +
cg04311230	6	159,693,649	*SOD2*	TSS1500	4.52(6.68,2.78)	7.40E-06	–	–	–	–	–
cg04349420	8	48,501,719		IGR	4.73(6.66,2.76)	2.79E-06	–	–	–	–	–
cg07797073	1	2,051,352	*PRKCZ*	Body	4.71(− 6.61,− 2.71)	3.02E-06	–	–	–	–	–
cg11491381	20	3,082,857	*AVP*	Body	− 4.66(6.55,2.65)	3.85E-06	–	–	–	–	–
cg18862005	2	177,076,135		IGR	4.60(6.56,2.66)	5.10E-06	–	–	–	–	–
cg25794823	18	63,949,347	*HMSD*	TSS200	4.61(− 8.16,− 4.26)	4.90E-06	–	–	–	–	–

Summary statistics for the 44 CpG sites with nominal association (p value < 10^–6^) in the Discovery Analysis. Significant CpG sites in the meta-analysis are written in bold letters in the CpG site and p value columns. Numbers in italics in the p value column for the meta-analysis indicate CpG sites with more significant p values in the meta-analysis compared with the Discovery Analysis

CpG: CpG site ID; CHR: chromosome where the CpG site is located; BP: specific chromosomal position for the CpG site; Gene: Gene annotation (from the Illumina Manifest File); Feature: Genomic location of the CpG site. It could reside in the 5’ untranslated region (UTR), between 0 and 200 nucleotides from the transcription start site (TSS), TSS200, or between 200 and 1500 nucleotides from the TSS, TSS1500. It could be also located in the body of the gene, in the 3’UTR or in an intergenic region (IGR); COEFF (UCI,LCI): Effect size for the association of the CpG site with the ∆NIHSS, with information for the upper (UCI) and lower (LCI) 95% confidence intervals; P: *p* value for the association of the CpG site with the ∆NIHSS

In bold are the significant CpG sites in the meta-analysis

We found a differential feature enrichment (*p* value = 9.23 × 10^–03^) when comparing nominal CpG sites (*n* = 44) with all the CpG sites included in the analysis (*n* = 423,156) (Additional file 1: Table SIV). Specifically, we found that nominal CpG sites that were hypermethylated in association with ΔNIHSS tend to be located in the body of genes rather than in IGR. Hypomethylated nominally CpG sites were found with higher probability in the TSS200 in comparison with all the CpG sites analysed. The differential feature enrichment could suppose different effects in gene expression.

The DMR analysis revealed 50 regions associated (*p* value < 0.05) (Additional file 1: Table SV) with ΔNIHSS, and the DMB analysis showed a total of 323 blocks associated with ΔNIHSS (Additional file 1: Table SVI).

### Replication stage

All the CpG sites with a nominal *p* value (< 10^–06^) in the Discovery Analysis (*n* = 44) were evaluated in the Replication Cohort. The median ΔNIHSS also had a positive value in this cohort (Table 1).

**Fig. 3:**
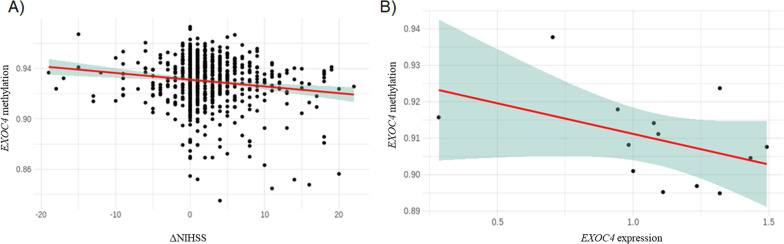
EXOC4 methylation in association with ΔNIHSS and with EXOC4 expression. **A** Correlation between EXOC4 methylation, calculated as *β* values of methylation (*Y*-axis) and ΔNIHSS (*X*-axis) for all the patients included in the Discovery Analysis. **B** Correlation between EXOC4 methylation, calculated as *β* values of methylation (*Y*-axis) and ΔEXOC4 expression (*X*-axis) assessed in 13 controls with EXOC4 expression and DNA methylation data

Six CpG sites could not be evaluated in the Replication Cohort because they fell to pass QCs (Table 2). From the 38 CpG sites that could be evaluated in the Replication Cohort, one site was significant (*p* value < 0.05): cg00039070 (*p* value = 1.14 × 10^–06^, coefficient = − 4.91 in the Discovery and *p* value = 1.14 × 10^–02^, coefficient = − 2.58 in the Replication Cohort) (Table 2). This CpG site was located in the body of the *EXOC4* gene, a member of the exocyst complex. The effect was the same in both cohorts: higher methylation was associated with stroke worsening measured with ΔNIHSS (Fig. 3A).

We analysed the association of the cg00039070 methylation with demographic and cardiovascular risk variables in the Discovery Cohort. We found sex, batch, PC1, PC2, baseline NIHSS and diabetes mellitus associated with cg00039070 methylation in bivariate analyses. After backward stepwise regression, only PC1 and PC2 remained significant. We re-analysed by lm the association of ΔNIHSS with cg00039070 methylation using only PC1 and PC2 as covariates. Methylation of cg00039070 in *EXOC4* maintained its association with ΔNIHSS with a *p* value of 2.27 × 10^–06^ and a coefficient of -4.77.

### EWAS meta-analysis

The meta-analysis combining effect sizes from the Discovery and Replication Cohorts revealed two CpG sites with epigenome-wide significant association, including the CpG site in *EXOC4* (*p* value = 4.44 × 10^–08^, coefficient = − 5.47), and 12 CpG sites with nominal association.

(Table 2). From these, all but one presented consistent effect direction in both Discovery and Replication analyses (Table 2).

The meta-analysis considering the ΔNIHSS as a dichotomic variable (ΔNIHSS ≥ 4 vs ΔNIHSS < 4) showed all but two CpG sites from the 38 analysed in the meta-analysis to be significantly associated with ΔNIHSS (*p* value < 0.05). The CpG site in *EXOC4* was also associated with the dichotomic ΔNIHSS with the same effect direction (*p* value = 4.56 × 10^–04^, coefficient: − 3.63) (Additional file 1: Table SVII).

### Pathway enrichment analysis

We identified different significantly enriched pathways after FDR adjustment (Q value < 0.05) using the methylRRA function from methylGSA but only one significant pathway (*p* value < 0.05) and any significant pathways when using methylglm and methylgometh, respectively (Table 3). The analyses were performed including as input the list of all the CpG sites (with the information of their *p* values) or the list of only the nominally associated CpG sites from the meta-analysis. As the feature enrichment analysis indicated an over-representation of CpG sites from the gene body and promoter regions, we decided also to test the pathway enrichment analysis depending on the CpG site location: gene body or gene promoter (TSS1500 and TSS200).

**Table 3:** Pathway enrichment analysis from meta-analysis EWAS results

Function	Database	Method	Sign CpG cut-off	Type CpGs	ID	Description	Size	*P* value	*Q* value
mglm	GO	N/A	No	Promoter	GO:0,030,100	Regulation of endocytosis	242	6.38E-04	5.36E-01
methylRRA	GO	GSA	Nominal	All	GO:0,016,358	Dendrite development	206	3.76E-04	1.62E-02
methylRRA	GO	GSA	Nominal	All	GO:0,048,588	Developmental cell growth	205	1.28E-03	2.75E-02
methylRRA	GO	GSA	No	All	R-HSA-5688426	Deubiquitination	206	1.83E-02	1.83E-02
methylRRA	Reactome	GSA	No	Promoter	R-HSA-5688426	Deubiquitination	206	4.57E-03	1.83E-02
methylRRA	Reactome	GSA	Nominal	Promoter	R-HSA-5688426	Deubiquitination	206	4.72E-03	1.89E-02
methylRRA	Reactome	ORA	Nominal	All	R-HSA-211859	Biological oxidations	203	3.08E-02	3.18E-02
methylRRA	Reactome	ORA	Nominal	All	R-HSA-72203	Processing of Capped Intron-Containing Pre-mRNA	203	3.08E-02	3.18E-02
methylRRA	Reactome	ORA	Nominal	All	R-HSA-162906	HIV infection	205	3.11E-02	3.18E-02
methylRRA	Reactome	ORA	Nominal	All	R-HSA-68882	Mitotic anaphase	209	3.17E-02	3.18E-02
methylRRA	Reactome	ORA	Nominal	All	R-HSA-2555396	Mitotic metaphase and anaphase	210	3.18E-02	3.18E-02
methylRRA	KEGG	ORA	Nominal	All	4510	Focal adhesion	200	3.03E-02	3.05E-02
methylRRA	KEGG	ORA	Nominal	All	4144	Endocytosis	201	3.05E-02	3.05E-02

Description of the significant pathways obtained from MethylGSA analysis using EWAS results from the meta-analysis

Function: Indicates which of the three MethylGSA functions was used; Database: The pathway database used in the analysis; Method: Method used (ORA or GSA) when methylRRA was selected; Sign CpG cut-off: “No” indicates that the analysis was performed including the results from all the CpG sites and “Nominal” indicates that the analysis was performed using the nominally associated CpG sites; type CpGs: “All” indicates that the analysis was performed for all the CpG types, while “promoter” indicates that in the analysis only CpG sites from promotors were analysed; ID: Identifier for the specific pathway from each database; Description: Detail of the pathway; Size: number of genes included in the gene set; and p value: enrichment p value for each gene set; Q value: FDR corrected *p *value

The endocytosis pathway was found to be enriched using the methylglm function (in the Gene Ontology database) and the methylRRA function (in the KEGG database). This pathway was enriched when all the CpG sites were considered but also when only the suggestive CpG sites were included. When CpG sites were filtered out based on their location, the endocytosis pathway was significant in two situations: when all the CpG sites were included independently of their location and when CpG sites from the promoter regions were selected (Table 3). The deubiquitination pathway was also found to be enriched in different situations with the methylRRA function: 1) using Gene Ontology and Reactome databases; 2) including all the CpG sites and only the nominal CpG sites; and 3) including only CpG sites in promoters but also with all the CpG site types (Table 3). Different pathways involving cell cycle and development were also overrepresented (Table 3).

### Gene expression and proteomic analyses

Only one CpG site (cg00039070 in *EXOC4*) met the pre-established criteria for being considered significant. This CpG site was located in the gene body, 10 kb downstream a predicted enhancer. Given that the effect of gene expression in gene body is variable, we wanted to assess whether this CpG site was affecting *EXOC4* expression. We found a negative correlation between cg00039070 methylation and *EXOC4* mRNA levels (Spearman correlation: -0.469) although it was not significant (*p* value = 0.091) (Fig. 3B).

To study the effect of *EXOC4* CpG site methylation at the proteomic level, we evaluated the proteins differentially expressed by *EXOC4* methylation using SOMAscan array data from 46 subjects (Additional file 1: Table SVIII). We found 79 differentially expressed proteins (*p* value < 0.05) in association with cg00039070 methylation. The most significant associations were for IFNA7 (*p* value = 8.52 × 10^–04^) and C8A, C8B, C8G (*p* value = 1.67 × 10^–03^) (Table 4). The pathway enrichment analysis using WebGestalt showed that the most significantly associated pathway related to significant proteins was NK cell activation (*p* value = 7.13 × 10^–04^) (Table 5).

**Table 4:** SOMAscan results

Protein	*t* value	*P* value
IFNA7	− 3.59	8.52 × 10^–04^
C8A.C8B.C8G	− 3.35	1.67 × 10^–03^
BMP1	− 3.09	3.52 × 10^–03^
IGFBP4	− 2.85	6.73 × 10^–03^
IL17A	− 2.60	1.28 × 10^–02^
CGA.FSHB	− 2.57	1.36 × 10^–02^
TGFB1	− 2.55	1.43 × 10^–02^
IFNL1	− 2.51	1.58 × 10^–02^
LCN2	− 2.49	1.66 × 10^–02^
SMAD3	− 2.48	1.70 × 10^–02^
BMPER	− 2.47	1.74 × 10^–02^
SERPINE2	− 2.46	1.78 × 10^–02^
IL23R	− 2.44	1.89 × 10^–02^
IL18R1	− 2.44	1.90 × 10^–02^

Summary statistics for the top significant *p* value < 0.01) association of EXOC4 methylation and protein levels measured by SOMAscan® Assay

**Table 5:** Pathway enrichment analysis for significant proteins associated with *EXOC4* methylation

Gene Set	Description	Size	Expect	Ratio	P value
GO:0,030,101	NK cell activation	85	0.21814	27.505	7.13 × 10^–04^
GO:0,042,110	T cell activation	452	11.600	8.6207	1.40 × 10^–03^
GO:0,050,673	Epithelial cell proliferation	372	0.95468	9.4272	3.10 × 10^–03^
GO:0,002,521	Leukocyte differentiation	496	12.729	7.8560	3.30 × 10^–03^
GO:0,070,661	Leukocyte proliferation	281	0.72115	11.093	4.54 × 10^–03^
GO:0,001,819	Positive regulation of cytokine production	418	10.727	8.3898	8.24 × 10^–03^
GO:0,002,250	Adaptive immune response	382	0.98035	8.1604	4.53 × 10^–06^
GO:0,002,285	Lymphocyte activation involved in immune response	172	0.44141	13.593	4.57 × 10^–06^
GO:0,018,212	Peptidyl-tyrosine modification	389	0.99831	8.0135	5.17 × 10^–06^
GO:0,002,449	Lymphocyte mediated immunity	238	0.61079	9.8233	2.9 × 10^–05^

Description of the significant pathways from Gene Ontology enriched among proteins significantly associated with ΔNIHSS methylation

Size: number of genes included in the gene set; expect: ratio of enrichment expected by chance in the gene set; Ratio: observed ratio for each specific gene set and p value: enrichment p value for each gene set

### DMCT analysis and tissue specificity analysis

We looked for differentially methylated cell types based on the 14 CpG sites significantly or nominally associated with ΔNIHSS in the meta-analysis. We only identified significant CpG sites in NK (in *NBEAL2* and *SLC7A6* genes) and B cells (in *NBEAL2* gene). However, the results were more significant in NK cells (*NBEAL2*: *p* value = 5.99 × 10^–09^*; SLC7A6*: *p* value = 8.65 × 10^–08^*)* than in B cells (*NBEAL2*: *p* value = 2.59 × 10^–07^) (Additional file 1: Figure SIII). There were not any CpG site in *EXOC4* differentially methylated in specific cell types.

We also looked for any tissue-specific regulatory component from the 14 CpG sites significantly and nominally associated with ΔNIHSS in the meta-analysis. Using as functional element the 15 chromatin state marks from the Roadmap project, we identified several brain tissue signals nominally enriched (*p* < 0.05), but not significant after multiple comparison adjustment in our results (Additional file 1: Table SIX).

### Blood and brain correlation

We looked for correlation in the cg00039070 methylation between blood and brain. Using the “Blood Brain DNA Methylation Comparison Tool”, we did not find any correlation in cg00039070 between any of the four brain regions included in this tool (prefrontal cortex, entorhinal cortex, superior temporal gyrus and cerebellum) and blood (Additional file 1: Figure SIV).

Using BECon, we found a negative correlation (− 0.48) between the cg00039070 methylation in blood and BA20 (in the temporal cortex). This correlation was classified in the highest correlation percentile (90%) (considering all the CpG sites included in the tool) (Additional file 1: Figure SV). The BA7 region (in the parietal cortex) presented an intermediate correlation (in the 50–75% percentile) (Additional file 1: Figure SV). We found a lack of correlation in the prefrontal cortex that was also observed using the “Blood Brain DNA Methylation Comparison Tool”.

## Discussion

In this study, our aim was to analyse whether the neurological course could be associated with epigenetic modifications. With this objective, we studied the genome-wide DNA methylation pattern associated with ΔNIHSS at discharge by EWAS. DNA methylation is probably the most studied epigenetic variation, consisting of the addition of a methyl group to a cytosine, mainly in the context of cytosines and guanines (CpG sites). We selected ΔNIHSS at discharge as the main variable in the EWAS because it was independently associated with Rankin at 3 months. The results from our EWAS suggest that the neurological course of stroke patients measured as the difference between NIHSS at baseline and NIHSS at discharge has an impact on DNA methylation in specific CpG sites.

From the 44 candidate CpG sites identified in the Discovery Analysis, two CpG sites, located in genes bodies, were epigenome-wide significant (*p* value < 2.4 × 10^–07^) in the meta-analysis of the Discovery and Replication cohorts, but only one CpG site (cg00039070) located in the body of the *EXOC4* gene accomplished all the pre-established criteria to be considered significant.

*EXOC4*, also known as *SEC8*, encodes for a subunit in the exocyst complex, a protein complex involved in the tethering of secretory vesicles to the plasma membrane [48]. Different functions are attributed to the exocyst complex, including but not limited to, exocytosis, cell growth cytokinesis and neuronal development [48, 49]. It is highly expressed in the brain and is enriched in axon growth cones and dendritic branches [48]. We found that the methylation pattern identified in association with stroke outcome was enriched for brain specific regulatory signals. We also investigated specifically the correlation of the methylation in cg00039070 between blood and brain using different tools. The results indicated that the effect of the *EXOC4* methylation in brain could be specific for some brain regions and its effect could be exacerbated in patients with stroke.

The pathway enrichment analysis showed that the regulation of DNA methylation in stroke outcome could be mediated by regulation of the endocytosis and the deubiquitination. The endocytosis is the process by which extracellular material is entered to the cell. This process has been showed to be interconnected with the exocytosis in the regulation of different processes such as cell polarity [50]. Gachet et al*.* [51] demonstrated the affectation of endocytosis in mutated *SEC8* (*EXOC4)* yeasts and a relationship with the cytokinesis process. Later, Jose et al. [52] described the exocyst complex as a key network hub which is regulating and coordinating both endocytosis and exocytosis and the balance among both processes.

The ubiquitin proteasome pathway is involved in the degradation of proteins and is key in the maintenance of the correct neuronal and synaptic function. After stroke, different pathological pathways are activated in response to the neuronal injury such as mitochondrial autophagy, oxidative stress and inflammatory response [53]. All these processes are related to the ubiquitin proteasome system. The specific role of the ubiquitin proteasome system in physiological and pathological processes after stroke is still in investigation but it has been suggested as a potential target for new drugs [53].

Higher methylation in cg00039070 from the *EXOC4* gene seems to be associated with a decrease in the expression of *EXOC4* gene, based on our gene expression results. The higher methylation in cg00039070 identified in patients with a worse stroke outcome could be mediated by the decrease in the *EXOC4* expression. Our proteomic analysis also indicated a decrease in different protein levels linked to higher cg00039070 methylation. The results from the pathway analysis from proteins differentially expressed linked with *EXOC4* methylation, suggested that the inflammatory pathway, regulated by NK cells, could be involved in the regulation of stroke outcome by methylation. The results from the differential methylation studied by cell type also supported the involvement of this pathway. It showed some associations for the CpG sites identified in our study, especially in NK cells. NK cells are innate immune cells that infiltrate ischaemic stroke lesions in human brains [54]. The function of NK cells is regulated by activation and inhibitory receptors located in the cell surface. Thus, the endocytosis is also important for the NK cell receptors trafficking which is key to modulate the dynamic function of NK cells [55].

The Sec8 protein, encoded by *EXOC4,* has been seen to control the synaptic targeting and the insertion of glutamate receptors in the synapsis, controlling the directional movement of glutamate receptors to the post-synaptic membrane [49]. Another possible hypothesis for the association of *EXOC4* methylation and stroke outcome is the affectation of the glutamate receptors dynamism. In stroke, the release of the glutamate neurotransmitter is associated with ischaemic cell death in a process known as excitotoxicity. Briefly, the glutamate neurotransmitter is increased because of the ischaemic insult [56] and over-activates two kind of glutamate receptors: the N-methyl-D-aspartate receptor (NMDAR) and the α-amino-3-hydroxy-5-methyl-4-isoxazolepropionic acid receptor (AMPAR) [56, 57]. Activation of the synaptic NMDAR leads to pro-survival signalling [58], while activation of extra-synaptic NMDAR induces a downstream neurotoxic cascade [59] that finally causes delayed neuronal death. Both NMDAR and AMPAR have been reported to be associated with the Sec8 subunit of the exocyst complex [49, 60], involved in the targeting of these receptors to the post-synaptic membrane [49, 60]. Considering that our results indicated that *EXOC4* methylation is associated with a decrease in the expression of *EXOC4* and with worse neurological course, we hypothesize that Sec8 could be regulating the trafficking of synaptic glutamate receptors related to cell survival in stroke [58].

Moreover, apart from the differential methylation identified in *EXOC4,* we found another gene, *GRM3,* nominally associated with ΔNIHSS in the discovery, that encodes glutamate metabotropic receptor 3, also involved in excitotoxicity processes. This gene was associated with memory impairment in a genetic study in Alzheimer disease patients [61].

A recently published GWAS has identified seven loci associated with stroke outcome measured through the NIHSS scale (calculating the difference between NIHSS at baseline and at 24 h) [13]. Their functional annotation strongly suggested *GRIA1* and *ADAM23* associated with ΔNIHSS. Both genes are also involved in excitotoxicity processes. Both results support a role of excitotoxicity in processes related to stroke neurological outcomes modulated by genetic and epigenetic variations. Despite clinical trials using drugs to modulate excitotoxicity processes having failed, progress has been made in clarifying the mechanisms that explain this failure [62].

One in vivo study in *EXOC4* mutant’s drosophila showed that apart from this gene being involved in glutamate receptor trafficking, it is also required for regulating synaptic microtubule formation and synaptic growth, thus suggesting that *EXOC4* methylation could be altering different processes in the synapsis [63].

The excitotoxicity, neuroinflammatory and the synaptic regulation are pathways that have been suggested to be pathological mediators of ischaemic brain damage [64] and could be potentially regulating the link between the methylation in *EXOC4* and the stroke outcome.

### Limitations

The first limitation is the difference in sample size and clinical features between the Discovery and Replication Cohorts. However, we looked for which clinical variables were associated with the methylation of *EXOC4* and we did not find any. Therefore, there is no reason to believe that they would affect *EXOC4* methylation in the Replication Cohort. Despite the differences between both cohorts, we have been able to replicate the results, which reinforces the plausible implication of *EXOC4* methylation in stroke outcome.

Another limitation is the use of whole blood to study DNA methylation in association with stroke outcome. However, other epigenomic, transcriptomic and proteomic studies on stroke [16, 65] have also used blood samples as it is also a relevant tissue in stroke outcome. Additionally, the blood and brain tissues have been found to have a 0.86 correlation in global methylation [66]. For that reason, we performed the analysis and then we correlated the methylation results in brain and blood tissues. Finally, we were not able to find a significant correlation between *EXOC4* mRNA levels and *EXOC4* methylation, despite a trend being observed. Probably, the sample size of for the transcriptomic analysis was not large enough to obtain significant results.

## Conclusions

We have expanded the knowledge about biological mechanisms regulating post-stroke outcome and highlighted the relevance of DNA methylation in explaining variability in functional outcome. Despite a small sample size, we had enough statistical power to obtain results that support the hypothesis of the excitotoxicity, neuroinflammatory and synapsis regulation pathways playing a significant role in stroke and indicate that further research is needed in this field to confirm this pathway as a future therapeutic target.

## Supplementary Information

**Additional file 1.** Supplemental Methods.

## Data Availability

The data sets used and/or analysed during the current study are available from the corresponding author on reasonable request.

## Abbreviations

AMPAR: α-amino-3-hydroxy-5-methyl-4-isoxazolepropionic acid receptor
DALYs: Disability-adjusted life-years
DMBs: Differential methylation blocks
DMCT: Differentially methylated cell types
DMP: Differentially methylated positions
DMRs: Differential methylation regions
EWAS: Epigenome-wide association study
GSA: Gene set enrichment analysis
GWAS: Genome-wide association studies
MRI: Magnetic resonance imaging
mRS: Modified Rankin Scale
NK: Natural killer
NMDAR: *N*-methyl-d-aspartate receptor
NIHSS: National Institutes of Health Stroke Scale
ORA: Over-representation analysis
PC: Principal components
QCs: Quality controls
TSS: Transcription start site
UTR: Untranslated region
