# Supplementary material for: Altered methylation pattern in EXOC4 is associated with stroke outcome: an epigenome-wide association study
Source: Clin Epigenetics. 2022 Sep 30;14:124. doi: 10.1186/s13148-022-01340-5 (PMC9526296; doi:10.1186/s13148-022-01340-5)
Supplement: Supplementary file 1 — Additional file 1. Supplemental Methods. [file 13148_2022_1340_MOESM1_ESM.docx]

**SUPPLEMENTARY MATERIAL**

**Altered methylation pattern in EXOC4 is associated with stroke outcome: An Epigenome-Wide Association study**

Natalia Cullell, MSc^1,2,3^, Carolina Soriano-Tárraga, PhD^4^, Cristina Gallego-Fábrega, PhD^1^, Jara Cárcel-Márquez, MSc^1^,Elena Muiño, MD^1^, Laia Llucià-Carol MSc ^1^, Miquel Lledós MSc ^1^,Manel Esteller, PhD^7,8,9^, Manuel Castro, PhD^7^, Joan Montaner, MD, PhD^5,10^, Anna Rosell, PhD^5^, Roberto Elosúa, MD^11^, Alba Fernández-Sanlés, PhD^11^, Joan Martí-Fábregas, MD, PhD^12^, Jerzy Krupinski, MD, PhD^2,6^ , Jaume Roquer, MD^4^, Jordi Jiménez-Conde, MD, PhD^4*^, Israel Fernández-Cadenas, PhD^1*^

1- Stroke Pharmacogenomics and Genetics, Institut de Recerca de Sant Pau, Hospital Sant Pau, Barcelona, Spain.

2- Neurology. Hospital Universitari MútuaTerrassa/ Fundacio Docència i Recerca MutuaTerrassa, Terrassa, Spain

3- Facultat de Medicina. Universitat de Barcelona, Barcelona, Spain

4- Neurology, Hospital del Mar; Neurovascular Research Group, IMIM; Universitat Autònoma de Barcelona / DCEXS‐Universitat Pompeu Fabra, Barcelona, Spain

5-Department of Psychiatry, Washington University School of Medicine, Saint Louis, Missouri, US

6-NeuroGenomics and Informatics, Washington University School of Medicine, Saint Louis, Missouri, US

7-Cancer Epigenetics & Biology Program (PEBC), L’Hospitalet, Barcelona, Spain

8-Department of Physiological Sciences II, School of Medicine, Universitat de Barcelona, Barcelona, Spain

9-Institució Catalana de Recerca i Estudis Avançats (ICREA), Barcelona, Spain

10- Neurovascular Research Laboratory, Vall d'Hebron Institut de Recerca (VHIR), Barcelona, Spain

11-Instituto de Biomedicina de Sevilla, IBiS/Hospital Universitario Virgen del Rocío / CSIC. Universidad de Sevilla & Department of Neurology, Hospital Universitario Virgen Macarena Sevilla, Spain

12- Neurology, Hospital de la Santa Creu i Sant, Barcelona, Spain

13- Centre for Bioscience, School of HealthCare Science, Manchester Metropolitan University, Manchester, UK

**1-Supplemental Materials and Methods**

*Metabolic pathway enrichment and feature enrichment analysis*

We looked for enrichment of metabolic pathways using as input all the CpG-sites and the CpG-sites that were nominally associated (p-value < 10^-06^) with ΔNIHSS in the meta-analysis. The analysis was done with MethylGSA (1), a R package specifically designed for pathway analysis from EWAS results. We applied the three functions from the package (all of them designed to adjust for the number of CpGs in each gene to reduce possible bias). Briefly, the first function, methylglm, performs a logistic regression using the number of CpG-sites per gene as covariate. We applied this function for all the CpG types but also restricting the analysis to CpG-sites from promoters (TSS1500 or TSS200) or located in gene bodies. This function does not allow to indicate a p-value threshold for CpG-sites to be included, so we did the analysis including the list of all the CpG-sites analyzed with their meta-analysis p-value. The second function, methylRRA, applies an over-representation analysis (ORA) or Preranked version of Gene Set Enrichment Analysis (GSA-Preranked) in the gene pathway analysis (after correcting for the different p-values in each gene using Robust Rank Aggregation). We performed the analysis with all the CpG-site types, with all the CpG-sites in promoters and with all the CpG-sites in gene bodies. The third function, methylgometh, uses the weighted resampling and Wallenius non-central hypergoemetric approximation to adjust for the number of CpGs in each gene. This function does not allow to select CpG-sites only from promoters or gene bodies to be analyzed. With the second and third function we included the list of all the CpG-sites but also the list of nominally associated CpG-sites from the meta-analysis. We included in the analyses the three available pathway databases in MethylGSA: Gene Ontology, KEGG and Reactome. We considered significant associations when the p-value was < 0.05 and the Q-value (False-Discovery rate (FDR) adjusted p-value) was < 0.05.

*Tissue-specific signal detection*

We used eFORGE (experimentally-derived Functional element Overlap analysis of ReGions from EWAS) (2) to estimate tissue specific signals from the significant and nominal results from the EWAS meta-analysis. Briefly, eFORGE looks for enrichment of functional elements in the target CpG-sites compared with background DMPs. This tool provides functional data from ENCODE, Roadmap Epigenomics and Blueprint project. All the three projects describe DNAse I hypersensitivity sites as functional element. Moreover, 15-state chromatin marks and histone 3 (H3) marks are available from the Roadmap Epigenomics project. We included analyses with all the different functional elements. A p-value < 0.05 was considered a nominal association and a Q-value < 0.05 according to FDR adjustment was considered statistically significant.

*Blood–Brain Epigenetic Correlation*

In order to compare the methylation from the significant findings in the meta-analysis between blood and brain, we used the Blood Brain DNA Methylation Comparison Tool (3). This tool correlates methylation level from the 450K Illumina array CpG-sites between blood and four brain regions (prefrontal cortex, entorhinal cortex, superior temporal gyrus and cerebellum). We analyzed the correlation of cg00039070 methylation and the four brain regions included in the tool.

Using *Blood–Brain Epigenetic Concordance* (BECon) (4), we also investigated the concordance in the cg00039070 methylation between three brain regions (Brodmann area (BA) 10, BA20 and BA7) and blood using the three metrics available in this tool: DNA methylation variability between samples in the specific CpG-site, correlation (Pearson correlation and percentile of correlation when comparing with all the CpG-sites) and the effect of cell composition adjustment in methylation.

*Blood–Brain Epigenetic Correlation*

In order to compare the methylation from the significant findings in the meta-analysis between blood and brain, we used the Blood Brain DNA Methylation Comparison Tool (46). This tool correlates methylation level from the 450K Illumina array CpG-sites between blood and four brain regions (prefrontal cortex, entorhinal cortex, superior temporal gyrus and cerebellum). We analyzed the correlation of cg00039070 methylation and the four brain regions included in the tool.

Using *Blood–Brain Epigenetic Concordance* (BECon) (47), we also investigated the concordance in the cg00039070 methylation between three brain regions (Brodmann area (BA) 10, BA20 and BA7) and blood using the three metrics available in this tool: DNA methylation variability between samples in the specific CpG-site, correlation (Pearson correlation and percentile of correlation when comparing with all the CpG-sites) and the effect of cell composition adjustment in methylation.

**2- Supplemental e-FIGURES**

**Figure I: Workflow for CpG-sites and sample QCs**

**
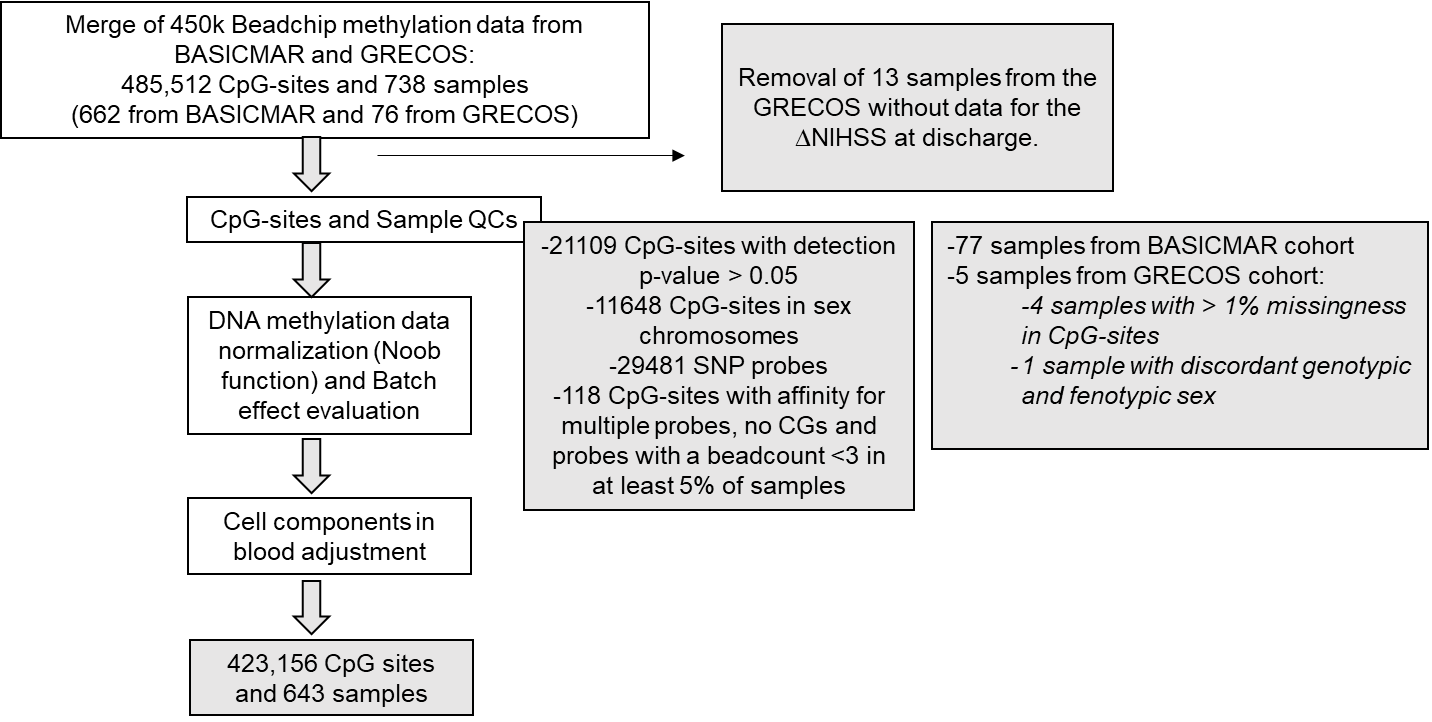
**

Description for the QCs applied to the Discovery cohorts.

**Figure II: Batch effect evaluation with MDS and SVD plots**

A) MDS plot stratifying samples with colours for batch effect (two different batch for the BASICMAR cohort and two batch for the GRECOS cohort) differentiation. Each colour identifies a different batch included in the Discovery cohort. B) Single Value Decomposition Analysis (SVD) to study the effect of principal component 1 and 2 (PC1 and PC2) and the batch (BASICMAR vs GRECOS cohorts) in our datasets.

**Figure III: Manhattan plots for DMCT**

Manhattan plot for the nominal CpG-sites in the meta-analysis stratified by cell-types: CD4+ T-cells, CD8+ T-cells, Neutrophiles, B-cells, Monocytes and natural killer (NK) cells. Green dots represent CpG-sites epigenome-wide associated with ΔNIHSS in the specific cell-type. Red and blue lines represent the epigenome-wide and nominal significance threshold, respectively.

**Figure IV: cg00039070 methylation correlation between blood and brain**

Correlation coefficients for the DNA methylation in cg00039070 between four brain regions and blood.

*PFC: prefrontal cortex; EC: entorhinal cortex; STG: superior temporal gyrus, CER: cerebellum*

**Figure V: Blood–Brain Epigenetic Concordance (BECon) results for cg00039070**

Interpretation of cg00039070 methylation in blood in the context of brain using BECon. Results are subdivided in three brain regions: Brodmann area (BA) 10, BA20 and BA7. Three different measures were used to assess the concordance between methylation in the *EXOC4* CpG-site methylation in blood and brain. 1^st^) Variability of the methylation in the CpG-site between the analysed samples in BECon. The colour annotation (described in the legend) indicates whether the CpG methylation is variable or not; 2^nd^) The Pearson correlation coefficient between the brain tissue and blood. The colour annotation indicates the correlation percentil of the specific CpG-site compared with all the CpG-sites analysed and 3^th^) The measure of the cell composition strength on the CpG in blood and brain. The colour shows how much the beta values for methylation change when adjusting by cell composition.

**3- Supplemental e-TABLES**

**Table I: Analysis of variables associated with ∆NIHSS in bivariate and regression analysis.**

|  | ∆NIHSS | |
| --- | --- | --- |
|  | **Bivariate analysis** | **Backward Stepwise Regression** |
| B_NIHSS | <2.2x10^-16*^ | <2.2x10^-16*^ |
| mRS 3 | 8.35x10^-07^* | <2.2x10^-16*^ |
| Age | 0.77 | - |
| Sex | 0.90 | - |
| B_glucose | 0.45 | - |
| DM | 0.09 | - |
| DL | 0.07 | - |
| tPA | 3.2x10^-09*^ | 2.5x10^-02^* |
| AF | 2.7x10^-04*^ | 1.75x10^-02^* |
| HTN | 0.46 | - |
| SMK | 0.13 | - |

∆NIHSS: difference between baseline NIHSS and NIHSS at discharge; B_NIHSS: NIHSS at baseline; B_glucose: glucose levels measured at baseline; DM: diabetes mellitus; DL: dyslipidemia; tPA: treatment with rtPA; AF: atrial fibrillation; HTN: hypertension; SMK; smoking habit.

**Table II: Analysis of variables associated with mRS at 3 months in bivariate and regression analysis.**

|  | mRS 3 | |
| --- | --- | --- |
|  | **Bivariate analysis** | **Backward Stepwise Regression** |
| ∆NIHSS | 8.35x10^-07^* | < 2x10^-16^* |
| B_NIHSS | <2.2x10^-16^* | < 2x10^-16^ |
| Age | <2.2x10^-16^* | 4.27x10^-10^ * |
| Sex | 9.11x10^-09^* | 1.3x10^-02^* |
| B_glucose | 1.2x10^-03^* | - |
| DM | 0.198 | - |
| DL | 4.7x10^-03^* | - |
| tPA | 0.6149 | - |
| AF | 7.67x10^-11^* | - |
| HTN | 0.08 | - |
| SMK | 4.098x10^-04^* | 4.4x10^-02^* |

∆NIHSS: difference between baseline NIHSS and NIHSS at discharge; B_NIHSS: NIHSS at baseline; B_glucose: glucose levels measured at baseline; DM: diabetes mellitus; DL: dyslipidemia; tPA: treatment with rtPA; AF: atrial fibrillation; HTN: hypertension; SMK; smoking habit.

**Table III: Summary statistics for the discovery EWAS adjusted by batch**

|  |  |  | Discovery (batch correction) | |
| --- | --- | --- | --- | --- |
| CpG | **CHR** | **BP** | **COEFF**  **(UCI.LCI)** | **P-value** |
| cg00039070 | 7 | 133482303 | -4.72(-6.67.-2.77) | 2.96E-06 |
| cg05979619 | 2 | 111947000 | -6.21(-8.15.-4.26) | 9.80E-10 |
| cg10977834 | 4 | 77075687 | 4.61(6.57.2.66) | 4.82E-06 |
| cg08119231 | 1 | 220747874 | 4.92(6.87.2.96) | 1.13E-06 |
| cg21574204 | 6 | 28081180 | 4.57(6.52.2.61) | 5.96E-06 |
| cg01149259 | 3 | 46979906 | 4.28(6.23.2.32) | 2.21E-05 |
| cg18831371 | 5 | 176610324 | 5.02(6.97.3.07) | 6.65E-07 |
| cg26050512 | 20 | 58981011 | 4.74(6.69.2.79) | 2.67E-06 |
| cg21404878 | 15 | 72286264 | 4.76(6.71.2.81) | 2.40E-06 |
| cg09548897 | 16 | 291277 | -4.69(-6.64.-2.74) | 3.34E-06 |
| cg14659930 | 3 | 114409454 | 4.43(6.38.2.48) | 1.10E-05 |
| cg00939347 | 10 | 606320 | -5.09(-7.04.-3.15) | 4.63E-07 |
| cg07925823 | 16 | 68264601 | 4.43(6.39.2.48) | 1.10E-05 |
| cg20383948 | 21 | 45478223 | 5.06(7.01.3.11) | 5.48E-07 |
| cg09741713 | 12 | 62078134 | 4.45(6.4.2.49) | 1.03E-05 |
| cg12103149 | 6 | 30213396 | 4.41(6.36.2.46) | 1.21E-05 |
| cg10156941 | 17 | 1563002 | 4.54(6.49.2.59) | 6.82E-06 |
| cg15765398 | 21 | 44990079 | -5.08(-7.03.-3.13) | 4.87E-07 |
| cg12349416 | 11 | 4184868 | 4.26(6.21.2.3) | 2.39E-05 |
| cg14482313 | 12 | 52233105 | 4.39(6.34.2.43) | 1.35E-05 |
| cg02996131 | 6 | 152637463 | 4.54(6.49.2.59) | 6.81E-06 |
| cg19935850 | 4 | 41751422 | 5.07(7.02.3.12) | 5.16E-07 |
| cg10363284 | 4 | 8005411 | -5.09(-7.04.-3.14) | 4.82E-07 |
| cg18707780 | 15 | 99733585 | 4.29(6.25.2.34) | 2.03E-05 |
| cg20648899 | 6 | 93416423 | 4.4(6.35.2.44) | 1.30E-05 |
| cg07987148 | 20 | 46690251 | 4.5(6.46.2.55) | 7.99E-06 |
| cg07475390 | 1 | 14114109 | 4.79(6.74.2.84) | 2.05E-06 |
| cg00347584 | 11 | 47261474 | 4.43(6.38.2.48) | 1.11E-05 |
| cg13114315 | 21 | 36174169 | 4.25(6.21.2.3) | 2.45E-05 |
| cg14414100 | 9 | 19547532 | 4.5(6.45.2.54) | 8.28E-06 |
| cg18795809 | 4 | 10456907 | 4.51(6.46.2.56) | 7.80E-06 |
| cg03732020 | 11 | 47261417 | 4.42(6.37.2.46) | 1.18E-05 |
| cg24978805 | 7 | 3687557 | -4.64(-6.59.-2.68) | 4.33E-06 |
| cg08526825 | 16 | 2752228 | 4.4(6.36.2.45) | 1.26E-05 |
| cg25354926 | 4 | 818823 | -4.24(-6.2.-2.29) | 2.54E-05 |
| cg04886221 | 1 | 27343238 | 4.43(6.39.2.48) | 1.09E-05 |
| cg22363670 | 7 | 86643853 | 4.34(6.3.2.39) | 1.64E-05 |
| cg06933752 | 11 | 65122316 | 4.99(6.94.3.04) | 7.94E-07 |
| cg04311230 | 6 | 159693649 | 2.55(4.51.0.58) | 0.011083 |
| cg04349420 | 8 | 48501719 | 4.34(6.3.2.39) | 1.63E-05 |
| cg07797073 | 1 | 2051352 | 4.66(6.62.2.71) | 3.81E-06 |
| cg11491381 | 20 | 3082857 | -3.91(-5.87.-1.95) | 0.000102 |
| cg18862005 | 2 | 177076135 | 3.8(5.75.1.84) | 0.000162 |
| cg25794823 | 18 | 63949347 | 4.34(6.29.2.38) | 1.68E-05 |

Summary statistics for the EWAS analysis adjusted by batch of the 44 CpG sites with nominal association (p-value < 10^-6^) in the Discovery Analysis .

*CpG: CpG site ID; CHR: chromosome where the CpG site is located; BP: specific chromosomal position for the CpG site; COEFF (UCI,LCI): Effect size for the association of the CpG-site with the ∆NIHSS, with information for the upper (UCI) and lower (LCI) 95% confidence intervals; P-value: p-value for the association of the CpG-site with the ∆NIHSS.*

**Table IV: Feature enrichment analysis**

|  | **EWAS analysis** | **Hypermethylation 🡪 ∆NIHSS > 0** | | | | | | | | **Hypomethylation 🡪 ∆NIHSS > 0** | | | | | | | | **Enrichement analysis** | **P_value** |
| --- | --- | --- | --- | --- | --- | --- | --- | --- | --- | --- | --- | --- | --- | --- | --- | --- | --- | --- | --- |
|  |  | 1stExon | 3'UTR | 5'UTR | Body | IGR | TSS1500 | TSS200 | 1stExon | | 3'UTR | 5'UTR | Body | IGR | TSS1500 | TSS200 |  | |  |
| DISCOVERY | SIGN. (N = 5) | 0.0% | 0.0% | 0.0% | 60.0% | 20.0% | 0.0% | 0.0% | 0.0% | | 0.0% | 0.0% | 0.0% | 0.0% | 0.0% | 20.0% | VS ALL | | 0.48 |
|  | NOMINAL (N = 44) | 0.0% | 0.0% | 0.0% | 18.2% | 2.3% | 0.0% | 0.0% | 2.3% | | 2.3% | 15.9% | 15.9% | 11.4% | 11.4% | 20.5% | VS ALL | | 9.23x10^-03^ |
|  | ALL (N = 423,156) | 1.7% | 1.5% | 3.2% | 13.8% | 10.1% | 5.2% | 3.5% | 3.3% | | 1.9% | 5.6% | 19.2% | 14.0% | 9.2% | 7.8% |  | |  |

Feature enrichment analysis considering the classification of hypermethylated and hypomethylated CpG-sites associated with ∆NIHSS improvement. Different feature comparisons were done depending on the CpG-sites considered.

The features for the significant (N = 5) and significant+nominal (n = 44) CpG-sites from the Discovery analysis were compared with the features from all the CpG-sites included in the array (after QCs; N = 423,156).

EWAS analysis column: analysis considered for CpG-site selection; Hypermethylation and Hypomethylation columns: percentage of CpG-sites belonging to each feature depending on their methylation status regarding ∆NIHSS. Enrichment analysis column: CpG-site background used for comparison (all CpG-sites or all nominal CpG-sites from the Discovery). P_value: p-value for the enrichment analysis.

*∆NIHSS: difference between baseline NIHSS and NIHSS at discharge; 3’UTR: 3’ untranslated region (UTR); 5’UTR: 5’ untranslated region (UTR); TSS200: transcription start site (TSS) between 0 and 200 nucleotides from the TSS; TSS1500: transcription start site (TSS) between 200 and 1500 nucleotides from the TSS; IGR: intergenic region*

**Table V: Differentially methylated region (DMR) results**

| Chr | Start | End | Value | Width | P-value |
| --- | --- | --- | --- | --- | --- |
| 7 | 27183133 | 27184521 | 0,015 | 1388 | 1,87E-04 |
| 20 | 36148699 | 36149455 | -0,012 | 756 | 7,46E-04 |
| 1 | 248100276 | 248100614 | 0,027 | 338 | 1,62E-03 |
| 6 | 30039132 | 30039801 | 0,015 | 669 | 2,06E-03 |
| 12 | 75784541 | 75785232 | 0,022 | 691 | 2,21E-03 |
| 22 | 22221599 | 22221878 | 0,028 | 279 | 2,29E-03 |
| 6 | 28956226 | 28956731 | 0,013 | 505 | 3,14E-03 |
| 17 | 77924582 | 77924733 | 0,025 | 151 | 3,66E-03 |
| 6 | 32847548 | 32847845 | 0,015 | 297 | 4,01E-03 |
| 6 | 33280052 | 33280518 | 0,012 | 466 | 4,79E-03 |
| 5 | 149546331 | 149546471 | 0,021 | 140 | 6,10E-03 |
| 6 | 32118295 | 32118457 | 0,015 | 162 | 6,21E-03 |
| 7 | 1080473 | 1081250 | -0,021 | 777 | 6,55E-03 |
| 7 | 56515510 | 56516255 | -0,023 | 745 | 8,83E-03 |
| 17 | 7832680 | 7833237 | 0,017 | 557 | 9,70E-03 |
| 15 | 31515750 | 31516481 | 0,018 | 731 | 9,85E-03 |
| 1 | 161008462 | 161008826 | -0,016 | 364 | 1,11E-02 |
| 19 | 13875014 | 13875329 | 0,017 | 315 | 1,27E-02 |
| 22 | 45809596 | 45810043 | 0,017 | 447 | 1,33E-02 |
| 18 | 61616369 | 61616718 | 0,016 | 349 | 1,42E-02 |
| 17 | 33759512 | 33760249 | 0,017 | 737 | 1,43E-02 |
| 7 | 27142535 | 27143287 | 0,015 | 752 | 1,54E-02 |
| 12 | 14926572 | 14927099 | 0,012 | 527 | 1,71E-02 |
| 18 | 14132207 | 14132366 | 0,017 | 159 | 1,93E-02 |
| 5 | 1594579 | 1594863 | 0,020 | 284 | 2,16E-02 |
| 20 | 50418952 | 50418959 | 0,014 | 7 | 2,16E-02 |
| 22 | 42394590 | 42394853 | 0,019 | 263 | 2,17E-02 |
| 6 | 29911334 | 29911558 | 0,017 | 224 | 2,20E-02 |
| 1 | 205818956 | 205819463 | 0,017 | 507 | 2,39E-02 |
| 15 | 81426347 | 81426820 | 0,016 | 473 | 2,62E-02 |
| 12 | 108078821 | 108079012 | 0,011 | 191 | 3,03E-02 |
| 1 | 230415225 | 230415668 | 0,017 | 443 | 3,15E-02 |
| 1 | 210000883 | 210001082 | 0,015 | 199 | 3,16E-02 |
| 11 | 70507825 | 70508410 | 0,018 | 585 | 3,30E-02 |
| 11 | 67383377 | 67383862 | 0,012 | 485 | 3,34E-02 |
| 7 | 27225811 | 27226329 | 0,014 | 518 | 3,34E-02 |
| 6 | 31734147 | 31734580 | 0,014 | 433 | 3,38E-02 |
| 5 | 78985484 | 78985592 | 0,012 | 108 | 3,51E-02 |
| 1 | 76189707 | 76189770 | 0,014 | 63 | 3,55E-02 |
| 20 | 61340827 | 61340885 | 0,018 | 58 | 3,69E-02 |
| 15 | 22833149 | 22833335 | -0,017 | 186 | 4,04E-02 |
| 12 | 29302016 | 29302035 | 0,013 | 19 | 4,10E-02 |
| 10 | 124638756 | 124639110 | 0,017 | 354 | 4,25E-02 |
| 3 | 45077369 | 45077920 | 0,014 | 551 | 4,46E-02 |
| 15 | 63340581 | 63340702 | 0,014 | 121 | 4,53E-02 |
| 17 | 17109936 | 17110353 | 0,016 | 417 | 4,60E-02 |
| 2 | 157184816 | 157184978 | 0,018 | 162 | 4,62E-02 |
| 20 | 36322069 | 36322169 | 0,013 | 100 | 4,92E-02 |
| 16 | 30441151 | 30441498 | 0,016 | 347 | 4,92E-02 |
| 15 | 41952827 | 41953061 | 0,017 | 234 | 4,92E-02 |

Significant DMR results (p-value < 0.05) in the Discovery cohort. Chr: chromosome where the DMR belongs to; Start-End: start position and final position of the DMR; Value: Average methylation variation in the DMR; Width: number of CpG-sites included in the DMR; P-value: p-value for the association of the DMR with ∆NIHSS.

**Table VI: Differentially methylated block (DMB) results**

| Chr | Start | End | Value | P-value |
| --- | --- | --- | --- | --- |
| 2 | 212609384 | 212618994 | 0,00351132 | 1,47E-05 |
| 2 | 213248884 | 213292541 | 0,00268735 | 4,30E-05 |
| 20 | 61158255 | 61167926 | -0,00114466 | 2,95E-04 |
| 4 | 92879355 | 92879355 | 0,00164698 | 5,85E-04 |
| 5 | 97645526 | 97728044 | -0,00103882 | 6,00E-04 |
| 22 | 22843648 | 22843648 | -0,00160559 | 6,60E-04 |
| 6 | 62596341 | 62737607 | -0,00120416 | 7,11E-04 |
| 13 | 67479146 | 67721697 | 0,0009132 | 8,03E-04 |
| 8 | 91227102 | 91411453 | 0,00117496 | 8,29E-04 |
| 9 | 71795999 | 71819910 | 0,00099709 | 2,03E-03 |
| 1 | 99774415 | 99774415 | -0,00120781 | 2,20E-03 |
| 22 | 21089054 | 21090192,5 | 0,00100978 | 3,18E-03 |
| 22 | 22874756,4 | 22918718 | 0,00089951 | 3,50E-03 |
| 3 | 21426059 | 21447688 | -0,00083278 | 3,61E-03 |
| 6 | 98744658 | 99007865 | 0,00077624 | 3,86E-03 |
| 11 | 89232216 | 89322779 | 0,00087646 | 3,97E-03 |
| 3 | 177778301 | 181556886 | -0,00031368 | 4,05E-03 |
| 18 | 22033229 | 22039676,5 | -0,00095528 | 4,09E-03 |
| 9 | 72158091 | 72347081 | 0,00094816 | 4,24E-03 |
| 20 | 48178928 | 48252667 | 0,00093139 | 4,62E-03 |
| 12 | 10208551 | 11662075 | -0,00029217 | 4,81E-03 |
| 5 | 97315689 | 97445281 | 0,00091453 | 5,02E-03 |
| 4 | 96051461 | 96102203 | 0,00078167 | 5,07E-03 |
| 5 | 91988522 | 92582155 | -0,00056859 | 5,52E-03 |
| 8 | 136439150 | 136803393 | -0,00061732 | 6,10E-03 |
| 4 | 187037287 | 187637871 | -0,00033556 | 6,29E-03 |
| 4 | 169418137 | 170328730 | -0,00034564 | 6,65E-03 |
| 7 | 19408343 | 21788417,5 | -0,0003552 | 6,70E-03 |
| 3 | 81533970 | 81816600 | -0,00073733 | 6,82E-03 |
| 16 | 34214005,7 | 34265361 | 0,00078642 | 6,86E-03 |
| 14 | 34385840 | 34488059 | 0,00064496 | 6,86E-03 |
| 3 | 171283606 | 172325619 | -0,0003173 | 7,05E-03 |
| 4 | 125660016 | 125857940 | 0,00071598 | 7,87E-03 |
| 4 | 119910700 | 120726319 | -0,00040147 | 7,87E-03 |
| 15 | 25414750,6 | 25726500 | -0,00029 | 8,05E-03 |
| 6 | 85298261 | 85445250 | 0,00062238 | 8,27E-03 |
| 7 | 81475720 | 81475720 | 0,00088104 | 8,30E-03 |
| 12 | 15501337 | 16512966 | -0,00038982 | 8,35E-03 |
| 11 | 27453034 | 28985617 | -0,00035097 | 8,38E-03 |
| 9 | 71669714 | 71691305 | -0,00081309 | 8,53E-03 |
| 4 | 163661836 | 163907607 | 0,00069887 | 8,85E-03 |
| 6 | 154406753 | 155432132 | -0,00033527 | 9,34E-03 |
| 6 | 143474125 | 144294847 | -0,00033565 | 9,60E-03 |
| 2 | 77747049 | 77856944 | -0,00068491 | 9,75E-03 |
| 8 | 87404176 | 87632363 | -0,00062299 | 9,77E-03 |
| 4 | 7750448 | 8145692 | -0,00026979 | 9,82E-03 |
| 4 | 36162678 | 36257279 | 0,00068168 | 9,97E-03 |

Top block results (p-value < 10^-04^) in the Discovery cohort. Chr: chromosome where the DMR belongs to; Start-End: start position and final position of the block; Value: Average methylation variation in the block; P-value: p-value for the association of the block with ∆NIHSS.

**Table VII: EWAS summary statistics in the meta-analyses for dichotomic ∆NIHSS.**

| CpG | CHR | BP | Gene | Feature | COEFF  (UCI,LCI) | P |
| --- | --- | --- | --- | --- | --- | --- |
| cg00039070 | 7 | 133167057 | EXOC4 | Body | -3.67(-3.66,-3.67) | 4.56E-04 |
| cg00347584 | 11 | 47283025 | NR1H3 | Body | 3.66(3.67,3.65) | 2.29E-04 |
| cg00939347 | 10 | 652260 | DIP2C | Body | -3.07(-3.07,-3.07) | 2.16E-03 |
| cg01149259 | 3 | 47021396 | NBEAL2 | 1stExon | 2.60(2.60,2.60) | 4.44E-03 |
| cg02996131 | 6 | 152958598 | SYNE1 | TSS1500 | 4.18(4.19,4.17) | 3.04E-05 |
| cg03732020 | 11 | 47282968 | NR1H3 | Body | 4.26(4.27,4.25) | 2.27E-05 |
| cg04886221 | 1 | 27669729 | SYTL1 | 5'UTR | 2.33(2.33,2.33) | 1.37E-02 |
| cg05979619 | 2 | 112704577 | MERTK | Body | -2.61(-2.61,-2.61) | 1.07E-02 |
| cg06933752 | 11 | 64889788 | FAU | TSS200 | 2.83(2.83,2.83) | 3.00E-03 |
| cg07475390 | 1 | 14440604 |  | IGR | 2.38(2.39,2.38) | 1.89E-02 |
| cg07925823 | 16 | 68298504 | SLC7A6 | 5'UTR | 2.75(2.75,2.75) | 2.65E-03 |
| cg07987148 | 20 | 45318890 | TP53RK | TSS1500 | 3.26(3.27,3.25) | 1.02E-03 |
| cg08119231 | 1 | 220921216 | MOSC2 | TSS1500 | 3.19(3.20,3.19) | 1.29E-03 |
| cg08526825 | 16 | 2802229 | SRRM2 | TSS200 | 2.50(2.50,2.50) | 1.80E-02 |
| cg09548897 | 16 | 341277 | AXIN1 | Body | -3.00(-3.00,-3.00) | 5.27E-03 |
| cg09741713 | 12 | 62471915 | FAM19A2 | 5'UTR | 2.90(2.91,2.90) | 3.82E-03 |
| cg10156941 | 17 | 1466296 | PITPNA | TSS200 | 5.00(5.00,5.00) | 4.93E-04 |
| cg10363284 | 4 | 8007138 | ABLIM2 | Body | -4.09(-4.08,-4.09) | 4.60E-05 |
| cg10977834 | 4 | 77996840 | CCNI | 5'UTR | 4.33(4.33,4.33) | 8.58E-06 |
| cg12103149 | 6 | 30181173 | TRIM26 | TSS200 | 3.00(3.00,3.00) | 1.21E-04 |
| cg12349416 | 11 | 4206098 |  | IGR | 3.53(3.53,3.52) | 4.36E-04 |
| cg13114315 | 21 | 37546467 | DOPEY2 | Body | 1.98(2.00,1.97) | 4.55E-02 |
| cg14414100 | 9 | 19547530 | SLC24A2 | Body | 2.28(2.29,2.27) | 2.22E-02 |
| cg14482313 | 12 | 52626889 | KRT7 | TSS200 | 4.25(4.26,4.24) | 2.79E-05 |
| cg14659930 | 3 | 114128301 | ZBTB20 | 5'UTR | 3.32(3.34,3.30) | 8.71E-04 |
| cg15765398 | 21 | 46409994 |  | IGR | -3.58(-3.57,-3.59) | 3.36E-04 |
| cg18707780 | 15 | 100273790 | LYSMD4 | TSS200 | 3.50(3.50,3.50) | 6.31E-05 |
| cg18795809 | 4 | 10458531 | ZNF518B | 5'UTR | 2.71(2.72,2.71) | 7.17E-03 |
| cg18831371 | 5 | 176037325 | GPRIN1 | TSS200 | 3.00(3.00,3.00) | 3.37E-03 |
| cg19935850 | 4 | 41753439 |  | IGR | 3.51(3.52,3.50) | 4.88E-04 |
| cg20383948 | 21 | 46898137 | COL18A1 | Body | 2.75(2.75,2.75) | 7.97E-03 |
| cg20648899 | 6 | 94126141 | EPHA7 | Body | 2.95(2.96,2.93) | 3.28E-03 |
| cg21404878 | 15 | 72578605 | BRUNOL6 | 3'UTR | 2.08(2.08,2.07) | 3.66E-02 |
| cg21574204 | 6 | 28048958 | ZNF165 | 5'UTR | 4.00(4.00,4.00) | 3.32E-05 |
| cg22363670 | 7 | 86273169 | GRM3 | TSS200 | 1.73(1.73,1.73) | 6.79E-02 |
| cg24978805 | 7 | 3727189 | SDK1 | Body | -2.44(-2.43,-2.44) | 1.37E-02 |
| cg25354926 | 4 | 812611 | CPLX1 | Body | -1.75(-1.75,-1.75) | 8.79E-02 |
| cg26050512 | 20 | 57556066 | TH1L | TSS1500 | 3.33(3.33,3.33) | 7.55E-05 |

Summary statistics for EWAS results in the meta-analysis considering ∆NIHSS as a dichotomic variable (only for the 38 CpG sites with nominal association in the Discovery EWAS analysis of continuous ∆NIHSS and available in the Discovery and Replication cohorts).

*CpG: CpG site ID; CHR: chromosome where the CpG site is located; BP: specific chromosomal position for the CpG site; Gene: Gene annotation (from the Illumina Manifest File); Feature: Genomic location of the CpG site. It could reside in the 5’ untranslated region (UTR), between 0 and 200 nucleotides from the transcription start site (TSS), TSS200, or between 200 and 1500 nucleotides from the TSS, TSS1500. It could be also located in the body of the gene, in the 3’UTR or in an intergenic region (IGR); COEFF (UCI,LCI): Effect size for the association of the CpG-site with the dichotomic ∆NIHSS, with information for the upper (UCI) and lower (LCI) 95% confidence intervals; P: p-value for the association of the CpG-site with the dichotomic ∆NIHSS.*

**Table VIII: Demographic and clinical data for the subjects included in the analysis with SOMAscan**

|  | **Stroke patients** | **Controls** |
| --- | --- | --- |
| Subjects(n) | 26 | 20 |
| Sex  *Male,n(%)*  *Female,n(%)* | 10 (38.5%)  16 (61.5%) | 11 (55%)  9 (45%) |
| Age in years, median (IQR) | 77 (13) | 64 (5) |
| Presence of HTN, n(%) | 12 (46.15%) | 20 (100%) |
| Smoking,n(%) | 3 (11.5%) | 3 (15%) |
| Treatment with rtPA, n (%) | 26 (100%) | N/A |
| TOAST  ***CES, n(%)***  ***LAS, n(%)***  ***SVS, n(%)***  ***Und, n(%)***  ***NA, n(%)*** | 16 (35.9%)  5 (27.8%)  0 (0%)  3 (3.9%)  2 (7.7%) | N/A  N/A  N/A  N/A  N/A |

Demographic and clinical features for subjects included in the proteomic analysis with SOMAscan.

IQR: interquartile range; HTN: Hypertension; CES: cardioembolic stroke; LAS: large artery stroke; SVS: small vessel stroke; und: undetermined stroke.

**Table IX: eFORGE analysis**

| Project | Data type | Cell | Tissue | P-value | Q-value |
| --- | --- | --- | --- | --- | --- |
| Roadmap Epigenomics | DHS | E109 Small Intestine | Small Intestine | 0.0265 | 1 |
|  | Chromatin mark: Enh | E071 Brain Hippocampus Middle | Brain | 0.0115 | 1 |
|  | Chromatin mark: BivFlnk | E081 Fetal Brain Male | Brain | 0.0143 | 1 |
|  | Chromatin mark: TxWk | E092 Fetal Stomach | Digestive | 0.0158 | 1 |
|  | Chromatin mark: TxWk | E090 Fetal Muscle Leg | Muscle | 0.0172 | 1 |
|  | Chromatin mark: Enh | E074 Brain Substantia Nigra | Brain | 0.0177 | 1 |
|  | Chromatin mark: Enh | E006 H1 Derived Mesenchymal Stem Cells | ES-derived | 0.0277 | 1 |
|  | Chromatin mark: TssAFlnk | E082 Fetal Brain Female | Brain | 0.0284 | 1 |
|  | Chromatin mark: TxWk | E063 Adipose Nuclei | Adipose | 0.0304 | 1 |
|  | Chromatin mark: TxWk | E061 Foreskin Melanocyte Primary Cells skin03 | Epithelial | 0.0307 | 1 |
|  | Chromatin mark: EnhG | E098 Pancreas | Pancreas | 0.0342 | 1 |
|  | Chromatin mark: TssAFlnk | E112 Thymus | Thymus | 0.0373 | 1 |
|  | Chromatin mark: Enh | E054 Ganglion Eminence derived primary cultured neurospheres | Neurosphere | 0.0408 | 1 |
|  | Chromatin mark: Enh | E052 Muscle Satellite Cultured | Muscle | 0.0469 | 1 |
|  | Chromatin mark: EnhG | E059 Foreskin Melanocyte Primary Cells skin01 | Epithelial | 0.0474 | 1 |
|  | H3K4me1 | E006 H1 Derived Mesenchymal Stem Cells | ES Cell | 0.0339 | 1 |
| ENCODE | DHS | hESCT0 | ES Cell | 0.0267 | 1 |
| Blueprint | DHS | CD14+ CD16- monocyte | Venous Blood | 0.0422 | 1 |
|  | DHS | CD14+ CD16- monocyte | Cord Blood | 0.0439 | 1 |

Significant associations in the eFORGE analysis to identify tissue specific signal from the significant and nominal associations from the EWAS metaanalysis. DHS from Roadmap, ENCODE and blueprint projects was selected in the analysis.

*DHS: DNase I hypersensitive sites; ES: Embryonic stem; Enh: Enhancer; BivFlnk: Flanking bivalent transcription start site (TSS)/Enh; TxWk: weakly transcribed states; EnhG: Genic Enhancer; TssAFlnk: Flanking Active TSS*

**4- Supplemental References**

1. Ren, X.; Kuan, P.F. MethylGSA: A Bioconductor Package and Shiny App for DNA Methylation Data Length Bias Adjustment in Gene Set Testing. Bioinforma. Oxf. Engl. 2019, 35, 1958–1959.

2. Breeze, C.E.; Reynolds, A.P.; van Dongen, J.; Dunham, I.; Lazar, J.; Neph, S.; Vierstra, J.; Bourque, G.; Teschendorff, A.E.; Stamatoyannopoulos, J.A.; et al. EFORGE v2.0: Updated Analysis of Cell Type-Specific Signal in Epigenomic Data. Bioinforma. Oxf. Engl. 2019, 35, 4767–4769

3. Hannon, E.; Lunnon, K.; Schalkwyk, L.; Mill, J. Interindividual Methylomic Variation across Blood, Cortex, and Cerebellum: Implications for Epigenetic Studies of Neurological and Neuropsychiatric Phenotypes. Epigenetics 2015, 10, 1024–1032

4. Edgar, R.D.; Jones, M.J.; Meaney, M.J.; Turecki, G.; Kobor, M.S. BECon: A Tool for Interpreting DNA Methylation Findings from Blood in the Context of Brain. Transl. Psychiatry 2017, 7, e1187
